# Genetic modification of inflammation- and clonal hematopoiesis–associated cardiovascular risk

**DOI:** 10.1172/JCI168597

**Published:** 2023-07-27

**Authors:** Zhi Yu, Trevor P. Fidler, Yunfeng Ruan, Caitlyn Vlasschaert, Tetsushi Nakao, Md Mesbah Uddin, Taralynn Mack, Abhishek Niroula, J. Brett Heimlich, Seyedeh M. Zekavat, Christopher J. Gibson, Gabriel K. Griffin, Yuxuan Wang, Gina M. Peloso, Nancy Heard-Costa, Daniel Levy, Ramachandran S. Vasan, François Aguet, Kristin G. Ardlie, Kent D. Taylor, Stephen S. Rich, Jerome I. Rotter, Peter Libby, Siddhartha Jaiswal, Benjamin L. Ebert, Alexander G. Bick, Alan R. Tall, Pradeep Natarajan

**Affiliations:** 1 Broad Institute of MIT and Harvard, Cambridge, Massachusetts, USA.; 2 Cardiovascular Research Center and Center for Genomic Medicine, Massachusetts General Hospital, Boston, Massachusetts, USA.; 3 Division of Molecular Medicine, Department of Medicine, Columbia University Irving Medical Center, New York, New York, USA.; 4 Department of Medicine, Queen’s University, Kingston, Ontario, Canada.; 5 Department of Medical Oncology, Dana-Farber Cancer Institute, Boston, Massachusetts, USA.; 6 Division of Cardiovascular Medicine, Department of Medicine, Brigham and Women’s Hospital, Harvard Medical School, Boston, Massachusetts, USA.; 7 Department of Medicine, Vanderbilt University Medical Center, Nashville, Tennessee, USA.; 8 Department of Laboratory Medicine, Lund University, Lund, Sweden.; 9 Department of Ophthalmology, Massachusetts Eye and Ear Institute, Boston, Massachusetts, USA.; 10 Department of Pathology, Dana-Farber Cancer Institute, Boston, Massachusetts, USA.; 11 Department of Pathology, Brigham and Women’s Hospital, Boston, Massachusetts, USA.; 12 Department of Biostatistics, Boston University School of Public Health, Boston, Massachusetts, USA.; 13 Department of Medicine, School of Medicine, Boston University, Boston, Massachusetts, USA.; 14 Framingham Heart Study, Framingham, Massachusetts, USA.; 15 Division of Intramural Research, National Heart, Lung, and Blood Institute (NHLBI), NIH, Bethesda, Maryland, USA.; 16 Department of Epidemiology, Boston University School of Public Health, Boston, Massachusetts, USA.; 17 Institute for Translational Genomics and Population Sciences, Department of Pediatrics, The Lundquist Institute for Biomedical Innovation at Harbor-UCLA Medical Center, Torrance, California, USA.; 18 Center for Public Health Genomics, University of Virginia, Charlottesville, Virginia, USA.; 19 Department of Pathology and Institute for Stem Cell Biology and Regenerative Medicine, Stanford University School of Medicine, Stanford, California, USA.; 20 Department of Medicine, Harvard Medical School, Boston, Massachusetts, USA.

**Keywords:** Cardiology, Genetics, Cardiovascular disease, Mouse models, Population genetics

## Abstract

Clonal hematopoiesis of indeterminate potential (CHIP) is associated with an increased risk of cardiovascular diseases (CVDs), putatively via inflammasome activation. We pursued an inflammatory gene modifier scan for CHIP-associated CVD risk among 424,651 UK Biobank participants. We identified CHIP using whole-exome sequencing data of blood DNA and modeled as a composite, considering all driver genes together, as well as separately for common drivers (*DNMT3A*, *TET2*, *ASXL1*, and *JAK2*). We developed predicted gene expression scores for 26 inflammasome-related genes and assessed how they modify CHIP-associated CVD risk. We identified *IL1RAP* as a potential key molecule for CHIP-associated CVD risk across genes and increased *AIM2* gene expression leading to heightened *JAK2*- and *ASXL1*-associated CVD risk. We show that CRISPR-induced *Asxl1-*mutated murine macrophages had a particularly heightened inflammatory response to AIM2 agonism, associated with an increased DNA damage response, as well as increased IL-10 secretion, mirroring a CVD-protective effect of *IL10* expression in *ASXL1* CHIP. Our study supports the role of inflammasomes in CHIP-associated CVD and provides evidence to support gene-specific strategies to address CHIP-associated CVD risk.

## Graphical abstract

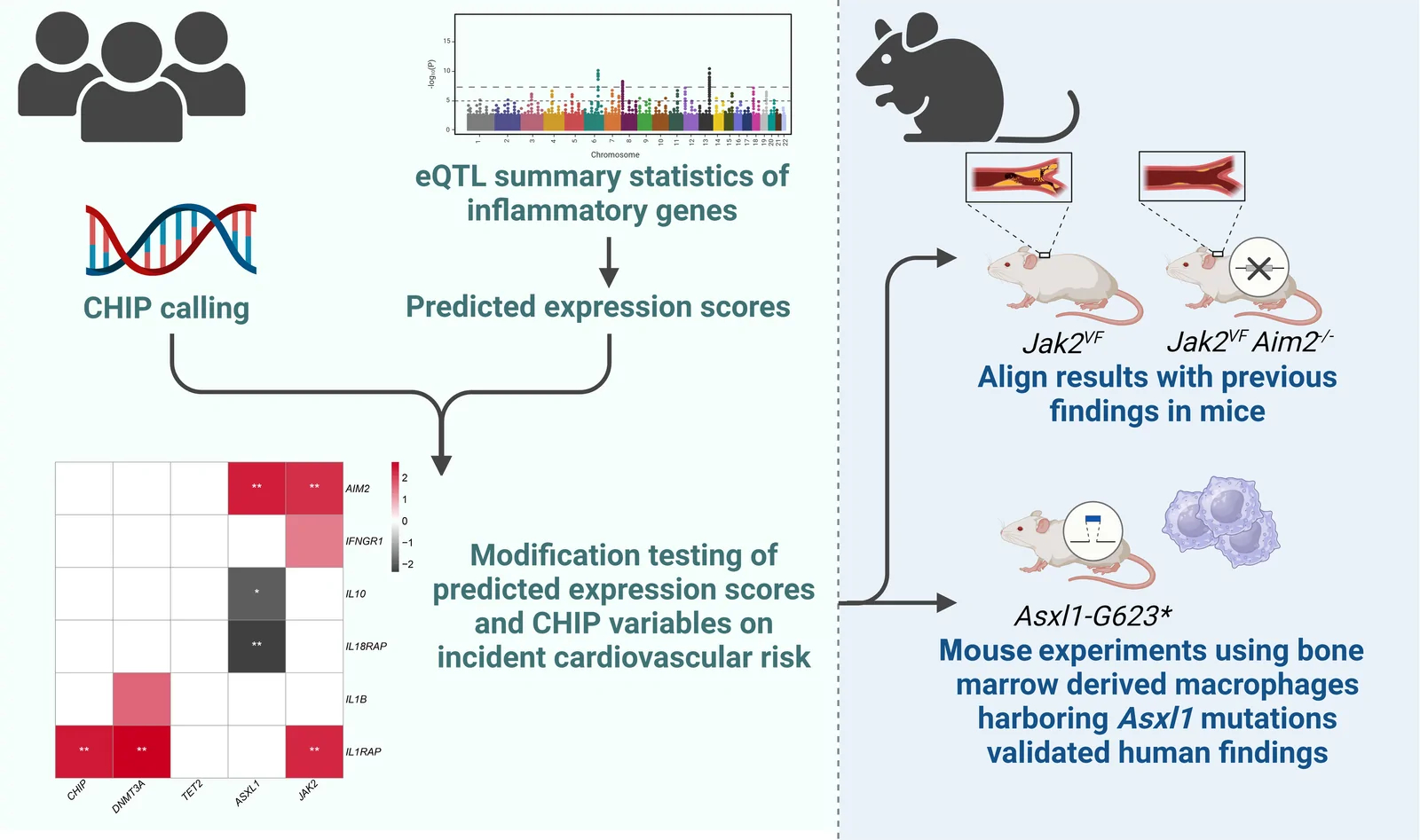

## Introduction

Clonal hematopoiesis (CH) of indeterminate potential (CHIP) is the age-related acquisition and expansion of somatic mutations of genes frequently mutated in hematologic malignancies (e.g., *DNMT3A*, *TET2*, *ASXL1*, or *JAK2*) (1) detected by sequencing blood DNA among asymptomatic individuals. CHIP is common among older adults, affecting at least 1 in 10 adults over 70 years (2–5). CHIP is associated with an increased risk of hematologic malignancy and all-cause mortality (3, 4), as well as a range of cardiovascular diseases (CVDs) (6–10). Recent evidence, primarily from murine and cell-based studies, suggests that dysregulated inflammation may be a key contributor to the augmented risk of CVD conferred by certain CHIP mutations (6, 11–14).

Heightened IL-1β signaling, a key inflammatory pathway, promotes the development of CHIP-associated atherosclerosis in *Tet2* CHIP as initially disclosed largely by murine studies (6, 11). Inhibition of the NOD-, LRR-, and pyrin domain-containing protein 3 (Nlrp3) inflammasome abrogates accelerated atherosclerosis in atherogenic mice with hematopoietic *Tet2* deficiency versus WT (11, 15). In humans, CHIP is associated with increased gene expression and circulating concentrations of NLRP3 downstream products, particularly in the context of *TET2*-mutant CHIP (*TET2* CHIP) (16–18). Individuals harboring *IL6R* p.Asp358Ala — a common variant known to disrupt *IL6R* and associate with modestly reduced CVD risk in the general population — had greater reductions in CVD risk when also carrying *DNMT3A* or *TET2* CHIP mutations versus those without (7). However, recent murine work indicates that different CHIP genes may confer CVD risk differentially. For example, among atherogenic transgenic mice expressing *Jak2^VF^*, bone marrow genetic deficiency of the absent in melanoma 2 (*Aim2*) inflammasome mitigated atherosclerotic lesion development (15). Whether these findings extend to humans is currently not well understood. In general, the range of inflammatory cytokines differentially influencing CVD risk by CHIP genes in humans requires further study. Prioritization by human genetics may yield or bolster new approaches to CVD precision medicine (19).

To overcome risks of confounding from biomarker correlation analyses, we leveraged genetics to pursue a broader inflammatory gene modifier scan for CHIP-associated CVD among 424,651 UK Biobank participants by performing blood DNA exome sequencing for CHIP genotyping; array-derived genome-wide genotyping for transcriptomic imputation; and assessment of baseline and incident clinical outcomes. We developed predicted gene expression scores for genes related to the NLRP3 and AIM2 inflammasomes based on externally trained data and conducted independent validation. Then we assessed whether and to what extent the predicted gene expression modifies CHIP-associated CVD risk. Last, we validated a human genomics–based discovery in a murine model. Broadly, we demonstrate a systematic approach to prioritizing potential therapeutic strategies for CHIP-associated disease.

## Results

### Baseline characteristics of the UK Biobank cohort.

**Figure 1:**
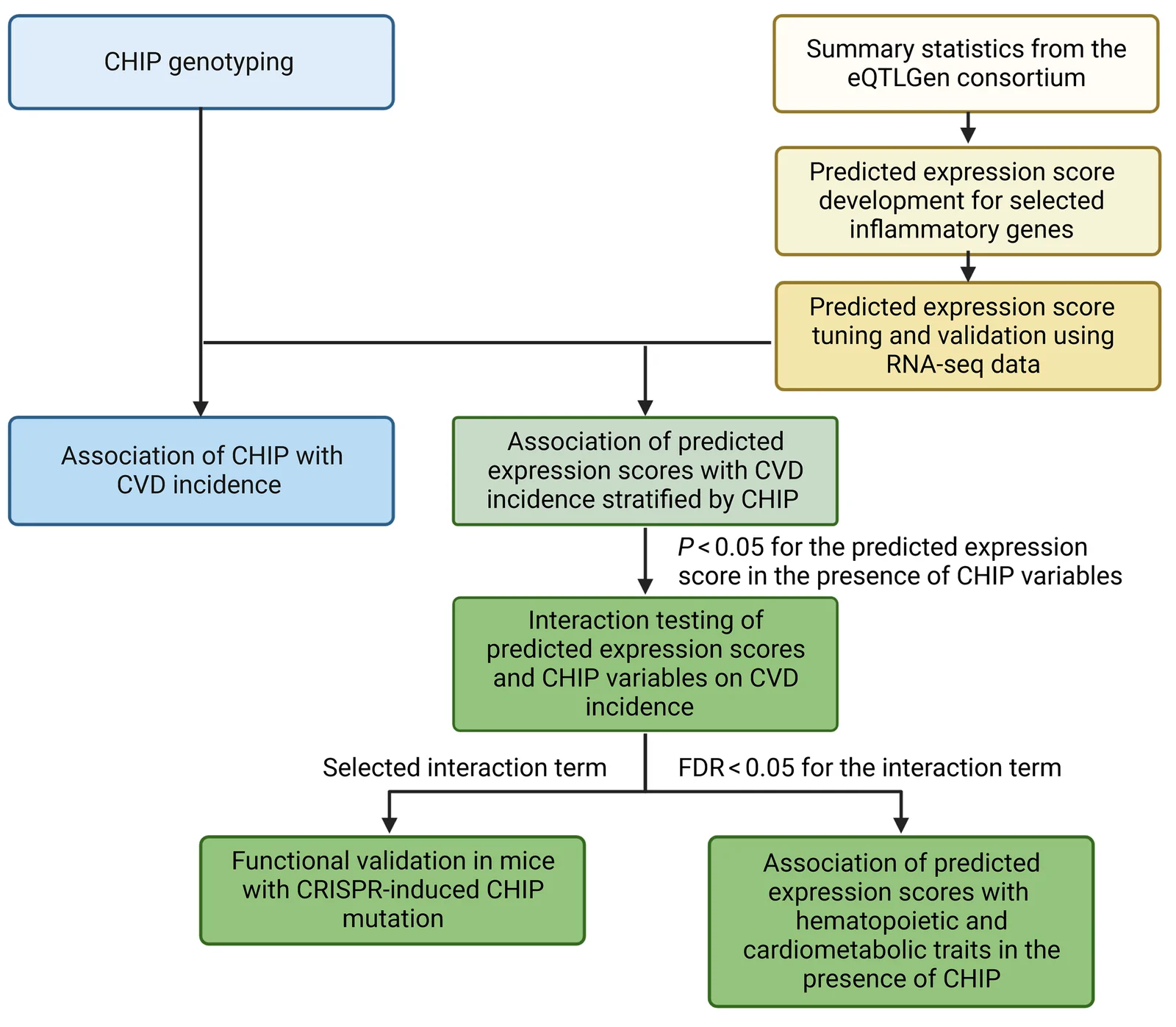
Study schematics. CHIP was identified using whole-exome sequencing data of blood DNA. Predicted expression scores for inflammatory genes were developed based on cis-eQTL results and validated using measured RNA-Seq data; we then examined whether they modified CHIP-associated CVD risk. Predicted expression scores that significantly modified CHIP-associated CVD risk were further validated in a mouse model and evaluated for their associations with hematopoietic and cardiometabolic traits.

The schematic of this study is shown in Figure 1. Among the 417,570 unrelated participants enrolled in the UK Biobank study who underwent exome sequencing and were free of hematologic cancers and composite CVD events at baseline, the mean age was 56.3 (SD 8.1) years, and 185,492 (44.4%) were men and 286,078 (55.6%) were women. We identified 25,784 (6.2%) individuals with CHIP mutations, with a mean age of 59.7 (SD 7.1). Among participants with CHIP mutations, 92.6% had only 1 driver mutation; 14,297 (55.4%) had mutations in *DNMT3A*, 5,133 (19.9%) in *TET2*, and 2,436 (9.1%) in *ASXL1*. Two hundred and forty-eight participants (1.0%) had *JAK2* mutations, 222 (89.5%) of whom had *JAK2* p.V617F and 241 (97.2%) had large clones, defined as having a variant allele fraction (VAF) of greater than 10%. Consistent with previous reports, participants with CHIP versus those without were on average 4 years older, were more likely to be White, had higher BMI, be ever-smokers, and had a higher prevalence of cardiovascular comorbidities, including hypertension, hyperlipidemia, and type 2 diabetes mellitus (Table 1).

### Associations between CHIP mutations and incident CVD.

During the 11.0-year median follow-up, 44,962 (10.6%) incident CVD events (a composite of myocardial infarction, coronary artery disease [CAD] or revascularization, stroke, or death (7) were observed. The presence of composite CHIP associated with increased CVD event risk independent of potential confounders (age, sex, White British ancestry, BMI at the time of enrollment, ever-smoker status, diagnosis of type 2 diabetes mellitus at the time of enrollment, and the first 10 principal components of genetic ancestry with a composite effect of HR 1.18 (95% CI: 1.14–1.22, *P* = 1.5 × 10^–21^). Among the top CHIP genes, CVD effects varied by gene, with *JAK2* 2.81-fold (95% CI 2.25–3.51, *P* = 8.5 × 10^–20^), *ASXL1* 1.41-fold (95% CI 1.29–1.54, *P* = 3.5 × 10^–14^), *TET2* 1.11-fold (95% CI 1.03–1.19, *P* = 4.5 × 10^–3^), and *DNMT3A* 1.06-fold (95% CI 1.01–1.11, *P* = 0.01). Other CHIP genes also showed significant associations with CVD incidence, with *SRSF2* 2.6-fold (95% CI 2.18–3.09, *P* = 6.8 × 10^–27^), *SF3B1* 1.47-fold (95% CI 1.14–1.89, *P* = 2.9 × 10^–3^), *TP53* 1.43-fold (95% CI 1.18–1.72, *P* = 2.2 × 10^–4^), and *PPM1D* 1.39-fold (95% CI 1.18–1.64, *P* = 7.6 × 10^–5^). Large clones generally demonstrated greater effects, with large CHIP associated with 1.29-fold (95% CI 1.24–1.35, *P* = 8.6 × 10^–29^) incident CVD risk (Table 2) (16). Sensitivity analyses restricting the outcome to CAD alone resulted in attenuated increases (Supplemental Table 1; supplemental material available online with this article; https://doi.org/10.1172/JCI168597DS1)

### Predicted expression of inflammatory genes.

We expanded the examination for CHIP modifiers through two dimensions: (i) In addition to a composite of all CHIP mutations at any driver genes, we examined the most commonly mutated CHIP genes individually (6), such as *DNMT3A*, *TET2*, *ASXL1,* and *JAK2*. (ii) In addition to *IL6R*, we generated predicted expression levels of all other inflammatory genes that are implicated in or closely related to the NLRP3 and AIM2 inflammasome pathways, including *NLRP3*, *IL1B*, *IFNG*, *IL18*, *CARD8*, *CASP1*, *CASP5*, *DHX33*, *IFNGR1*, *IFNGR2*, *IL1R1*, *IL1R2*, *IL1RAP*, *IL6*, *IL6ST*, *IL10*, *IL18BP*, *IL18R1*, *IL18RAP*, *IRF1*, *JAK1*, *JAK2*, *JAK3*, *NEK7*, *NLRC4*, *SOCS*, *STAT1*, *STAT3*, *STAT4*, *STAT5A*, *STAT6*, *TNF*, and *TYK2* (see Methods and Supplemental Figure 1).

**Figure 2:**
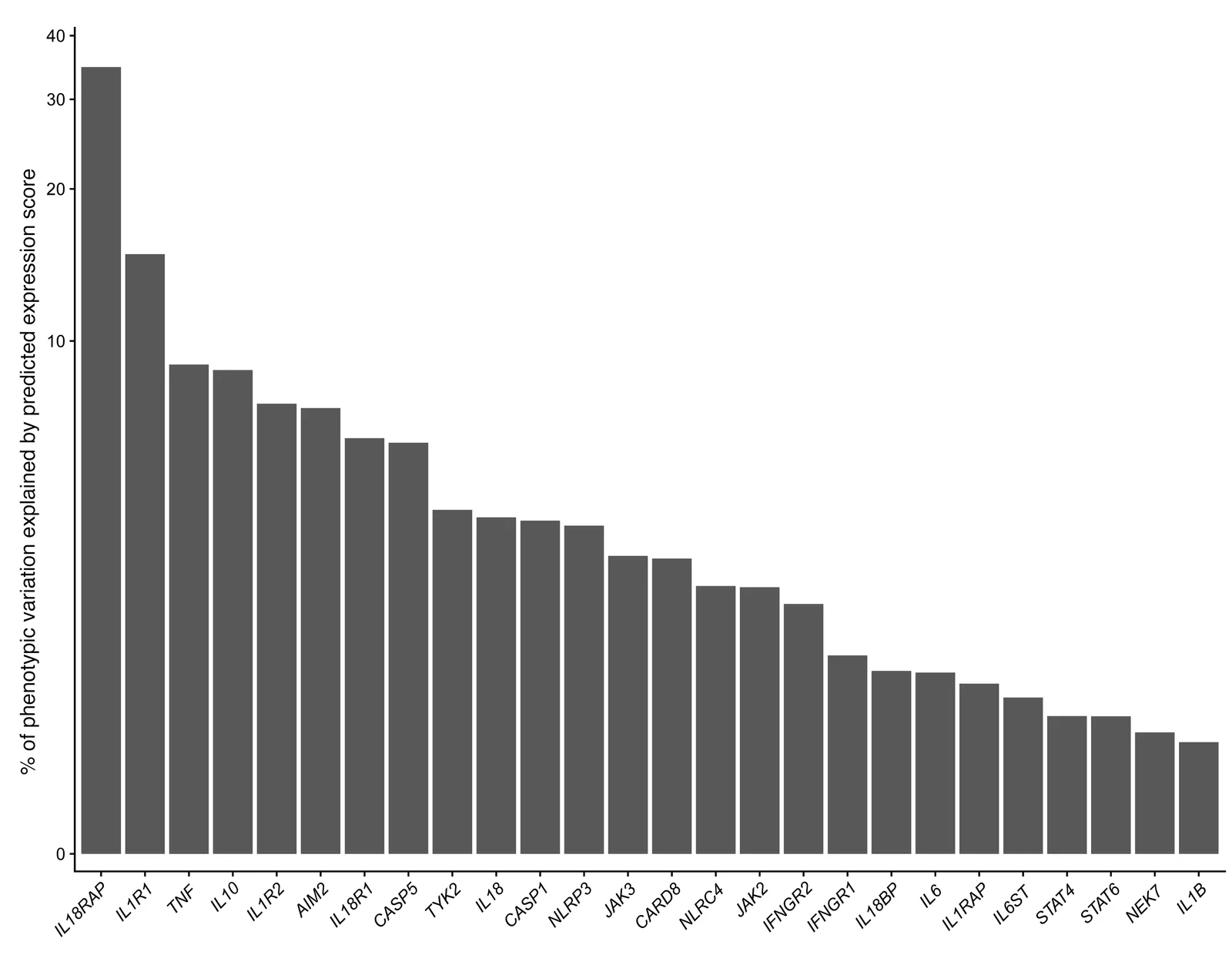
Proportion of the variance of experimentally measured expression levels that can be explained by predicted expression scores for inflammatory genes among participants with EA. Inflammatory genes were identified through canonical pathways and protein-protein interactions based on STRING. Predicted expression scores for examined genes were calculated by applying either the P+T or PRS-CS method to the summary statistics of the eQTL for those genes from the eQTLGen consortium (https://www.eqtlgen.org/) and validated using experimental measured RNA-Seq data in MESA (PBMCs) and FHS (whole blood). Since the eQTL source data were from either PBMCs or whole blood, we report the largest *r^2^* of the measured transcriptome levels in either FHS or MESA.

We developed predicted expression scores based on summary statistics of the whole-blood or PBMC *cis*-expression quantitative trait locus (eQTL) results for the corresponding genes from the eQTLGen Consortium (20). For each selected gene, we used both the pruning and thresholding (P+T) method (21) and the polygenic risk score–continuous shrinkage (PRS-CS) method (22) to generate a series of candidate scores for participants with European ancestry (EA) and non-EA separately; they were then tuned using nonoverlapping individual-level RNA-Seq data from the Framingham Heart Study (FHS; whole blood) and Multi-Ethnic Study of Atherosclerosis (MESA; PBMCs) (23, 24). The final predicted expression score of each gene was selected based on the proportion of the variance (*r^2^*) of experimentally measured expression levels that can be explained by the candidate scores (see Methods). For most genes, the P+T method generated a better score performance than PRS-CS (Supplemental Table 2). For this analysis, we continued studying genes whose selected best-performing predicted expression scores had *r^2^* > 1% among EA participants, resulting in scores for 26 (of 35 total evaluated) genes. The predicted expression scores explained a median of 3.5% (IQR 1.8%–6.3%) of the adjusted variance of corresponding gene expression levels among EA participants. The score for *IL18RAP* explained the largest proportion of phenotypic variance (34.7%), and that for *IL1B* explained the least variance (1.05%) among analyzed genes with *r^2^* > 1% (Figure 2 and Supplemental Table 2).

### Modification of CHIP-associated CVD risk by predicted expression of inflammatory genes.

**Figure 3:**
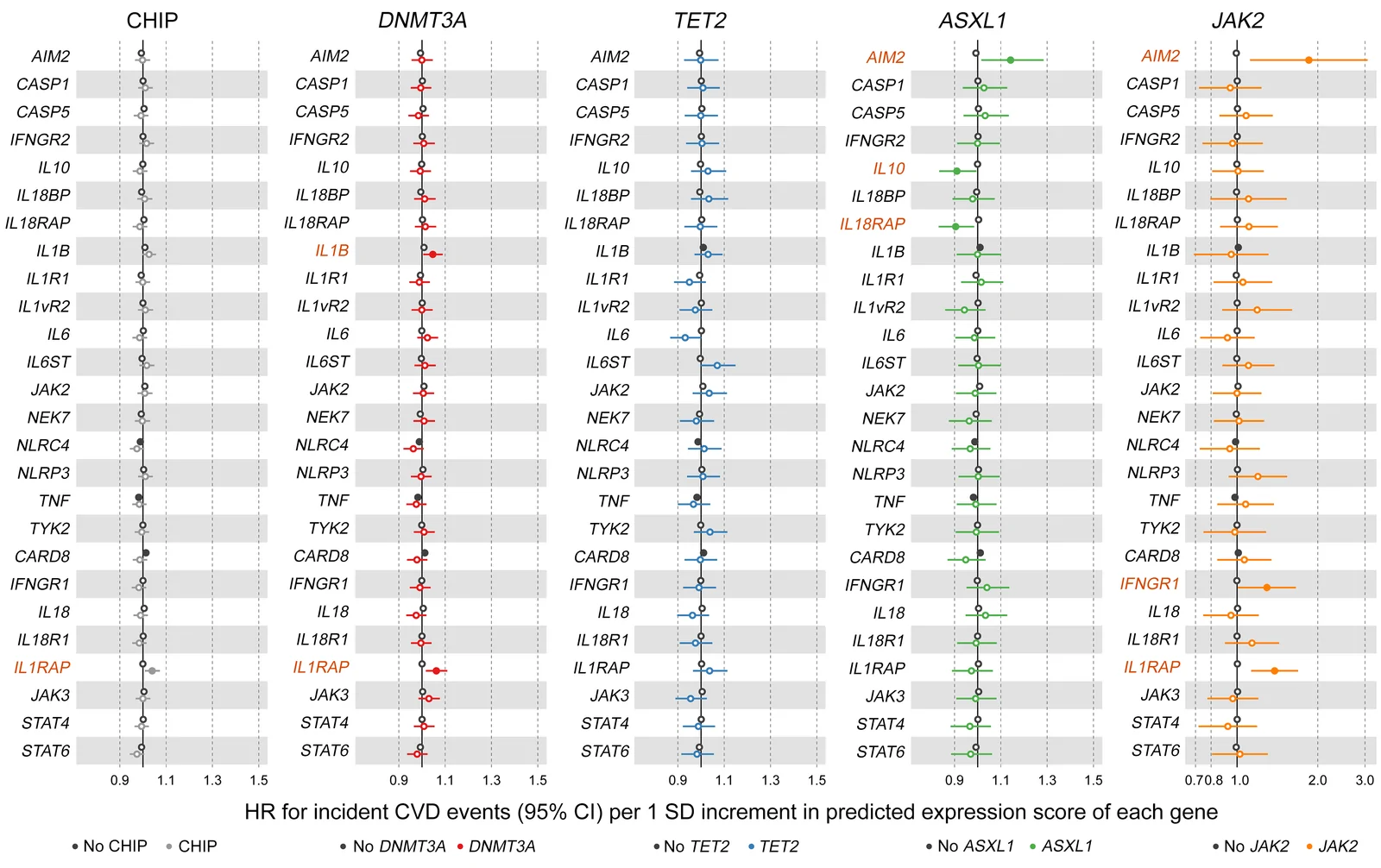
HR of 1 SD increment in predicted expression scores of inflammatory genes on CVD event incidence stratified by CHIP mutation status. Inflammatory genes were identified through canonical pathways and protein-protein interactions based on STRING. Predicted expression scores for examined genes were calculated by applying either the P+T or PRS-CS method to the summary statistics of the eQTL for those genes from the eQTLGen Consortium and validated using experimentally measured RNA-Seq data in MESA (PBMCs) and FHS (whole blood). CVD event outcome was defined as a composite of myocardial infarction, CAD or revascularization, stroke, or death. Black indicates the absence of CHIP mutations, and all other colors indicate the presence of CHIP mutations. Filled circles indicate a significant association at the *P* < 0.05 level. Red text for gene names indicates a significant association between the corresponding expression score and CVD outcome in the presence of CHIP mutation at the *P* < 0.05 level.

**Figure 4:**
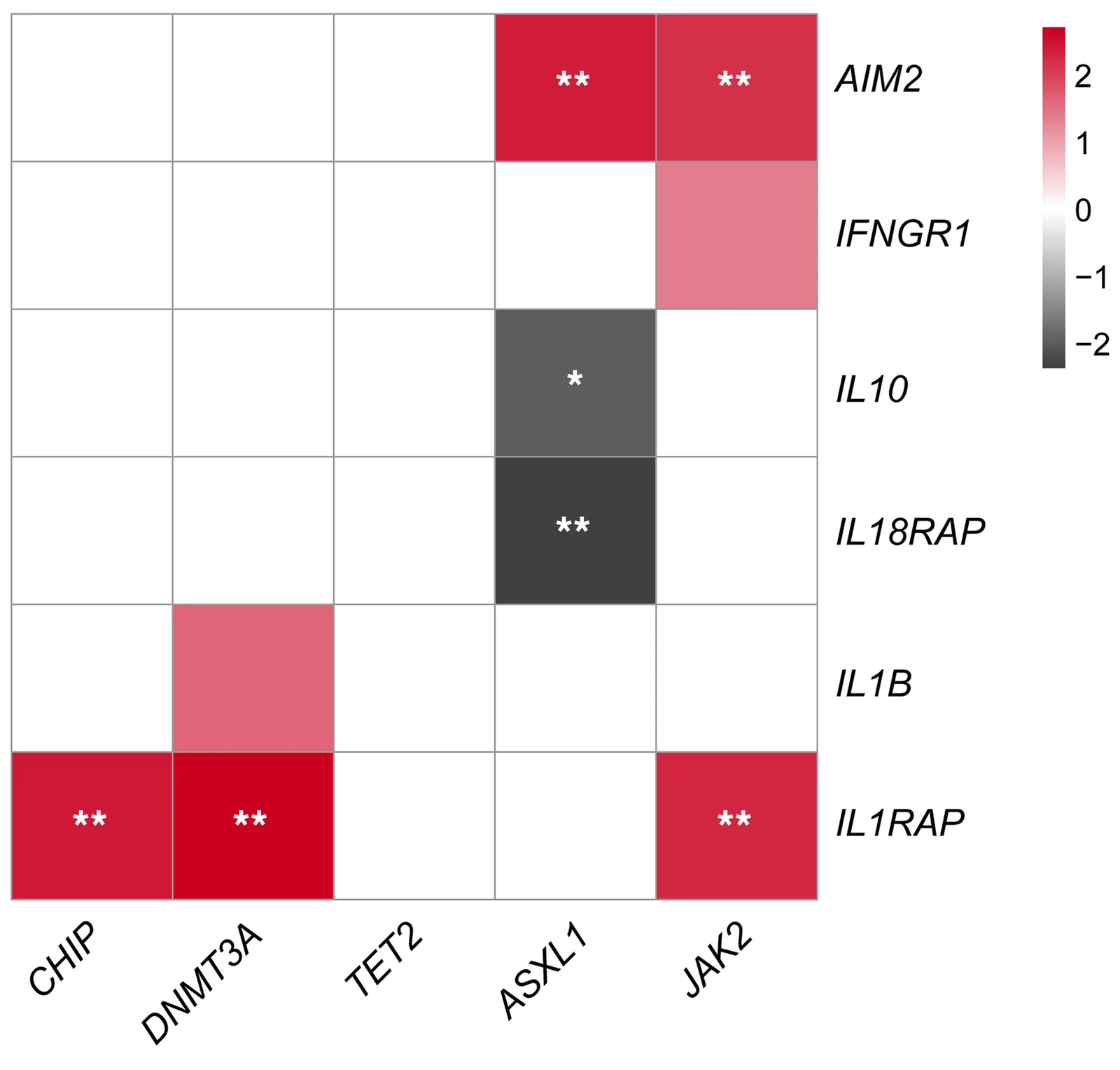
Heatmap for *z* scores of interactions between CHIP mutations and predicted expression scores of inflammatory genes on CVD event incidence. Only predicted expression scores significantly associated with CVD event incidence among participants with CHIP mutations were examined for their interactions in this step. Inflammatory genes were identified through canonical pathways and protein-protein interactions based on STRING. Predicted expression scores of examined genes were calculated by applying either the P+T or PRS-CS method to the summary statistics of the eQTL for those genes from the eQTLGen Consortium. CVD event outcome was defined as a composite of myocardial infarction, CAD or revascularization, stroke, or death. Black indicates a negative *z* score, and red indicates a positive *z* score. **Statistical significance at an FDR = 0.05 level; *statistical significance at an FDR = 0.1 level. The darker the color, the stronger the effects.

We observed significant associations between predicted expression scores of several inflammatory genes and incident CVD risk with the presence of CHIP or specific CHIP gene(s) (collectively called CHIP variables), while the corresponding associations for those without CHIP were all nonsignificant. We carried forward predicted expression scores that were significantly associated with incident CVD risk at a *P* < 0.05 level only in the presence of CHIP variable(s) to evaluate how the interactions between those scores and the corresponding CHIP variables (*n* = 9 pairs) associated with primary CVD outcome (Figures 3 and 4).

Regarding specific modification pairs, first we found evidence supporting recent murine findings (15) in humans in our observation that a genetic predisposition to higher *AIM2* expression was associated with amplified risk for incident CVD for those with *JAK2* CHIP. One SD increase in predicted expression score for *AIM2* was associated with an almost 2-fold increased risk in CVD incidence (HR 1.85, 95% CI 1.12–3.07, *P* = 0.02) among participants with *JAK2* mutations. In contrast, the predicted expression score for *AIM2* was not associated with incident CVD event risk in those without *JAK2* mutations (HR 0.99, 95% CI 0.98–1.00, *P* = 0.16), which was significantly different for those with *JAK2* CHIP (FDR for interaction, 0.04). Mice expressing *Jak2^VF^* in bone marrow had a 2-fold increase in atherosclerotic lesion development, which was reduced by genetic ablation of *Aim2* in mutant cells (15). Moreover, the CVD risk associated with *JAK2^VF^* CHIP was augmented by higher predicted expression of *IFNGR1*. IFN-γ increased *Aim2* expression in *Jak2^VF^* BMDMs, and AIM2 levels were increased in plaques of *Jak2^VF^* CHIP mice (15). These findings support the translational relevance of murine models of *Jak2^VF^* CHIP.

Second, we observed modification effects of the predicted expression level of *IL1RAP* on incident CVD risk associated with composite CHIP, *DNMT3A* CHIP, and *JAK2* CHIP mutations. *IL1RAP* encodes IL-1 receptor accessory protein (IL-1RAP), a coreceptor involved in several inflammatory signaling pathways and the lack of which completely abrogates cellular response to IL-1 (25–28). For 1 SD increase in the predicted expression score for *IL1RAP*, HRs were 1.04 (95% CI 1.01–1.07) in the presence of composite CHIP mutations, 1.06 (95% CI 1.02–1.11) in the presence of *DNMT3A* mutations, and 1.38 (95% CI 1.13–1.69) in the presence of *JAK2* mutations; in contrast with HRs of 1.00 (95% CI 0.99–1.01), 1.00 (95% CI 0.99–1.01), and 1.00 (95% CI 0.99–1.01) among participants without these mutations (FDR for interaction, 0.04, 0.04, and 0.04, respectively). While the relationship for *TET2* was directionally consistent, no significant association was observed. This result implicates IL1RAP as a potentially key IL-1β/IL-6 pathway–related molecule for CHIP-associated CVD risk across genes (7, 11).

Third, we identified potential modification effects of the predicted expression of *AIM2* and *IL10* on *ASXL1*-associated CVD risk. In addition to the effect of the *AIM2*’s aforementioned interaction with *JAK2* on CVD risk, predicted *AIM2* expression similarly modified *ASXL1*-associated CVD disease risk (*ASXL1* mutation present: HR 1.14, 95% CI 1.02–1.28; *ASXL1* mutation absent: HR 0.99, 95% CI 0.98–1.00; FDR for interaction, 0.04). Similar effects were not observed for *DNMT3A*- or *TET2*-associated CVD. *IL10* is expressed in atherosclerotic plaques, and its encoded protein, IL-10, is an antiinflammatory cytokine that inhibits many cellular processes that advance human atherosclerosis (29–37). The protective effect of IL-10 was pronounced in the presence of *ASXL1* mutation, with its predicted expression score associated with a significantly decreased risk of incident CVD (HR 0.91, 95% CI 0.83–0.99, *P* = 0.04) in the presence of *ASXL1* mutation but a null effect (HR 1.00, 95% CI 0.99–1.01, *P* = 0.91) in its absence (FDR for interaction, 0.06). Another molecule implicated was *IL18RAP*, which encodes IL-18RAP. IL-18RAP enhances the IL-18–binding activity of the IL-18 receptor and plays a role in signaling by the inflammatory cytokine IL-18 (38). However, we observed attenuated CVD risk associated with the predicted expression score of *IL18RAP* among participants with *ASXL1* mutation (HR 0.90, 95% CI 0.83–0.98, *P* = 0.02) but not those without (HR 1.00, 95% CI 0.99–1.01, *P* = 0.41; FDR for interaction, 0.04). These results are shown in Figures 3 and 4, and Supplemental Tables 3 and 4. These identified inflammatory expression scores that modify CHIP variable–associated CVD risk were not associated with the corresponding CHIP variable, with *JAK2* gene expression and *JAK2* CHIP mutation (FDR = 6.1 × 10^–6^) as the exception.

### AIM2 inflammasome activation in macrophages harboring Asxl1 mutations.

**Figure 5:**
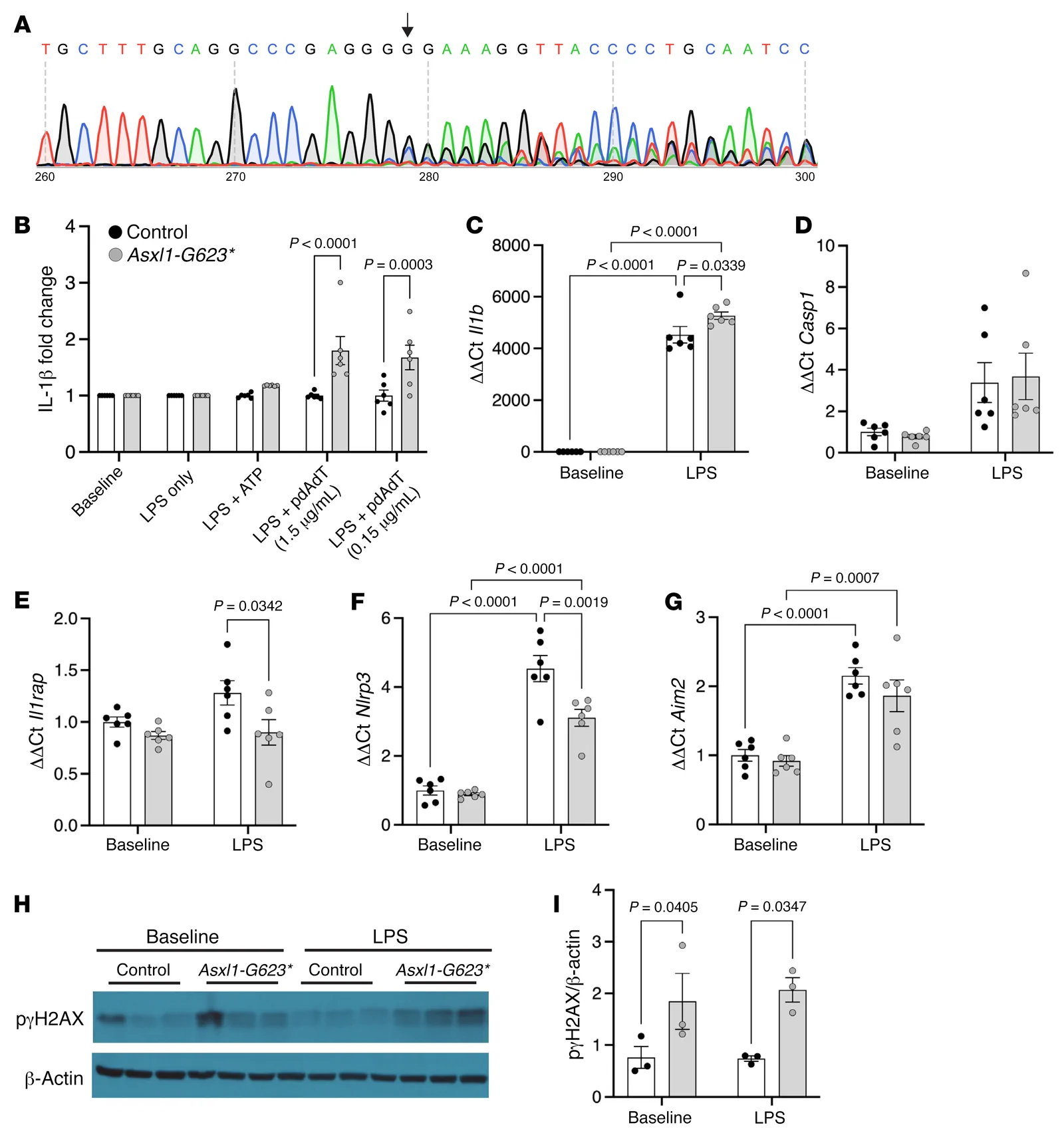
Inflammasome activation in BMDMs harboring *Asxl1* mutations. BMDMs were harvested from mice harboring a mixture of either WT control (Nmt4) or *Asxl1*-mutated bone marrow (Asxl1-G623*) and WT bone marrow. (**A**) Sanger sequencing of Cas9-transgenic murine fibroblasts transfected with lentiviruses containing Asxl1 guides targeting exon 12; arrow indicates target site. (**B**) Inflammasome activation was marked by IL-1β in supernatant of BMDMs primed with LPS, then ATP was used to stimulate NLRP3 inflammasome or pdAdT was used to activate the AIM2 inflammasome; data are presented as fold change. (**C**–**G**) qPCR analysis of BMDMs at baseline or following 6-hour stimulation with 20 ng/mL LPS. (**H**) Western blot analysis of BMDMs at baseline or following 6 hours of stimulation with 20 ng/mL LPS. (**I**) Densitometric quantification of the Western blot. Data are mean ± SEM. Two-way ANOVA followed by Tukey’s post hoc test, **B**–**G** and **I**.

Our findings indicated that the predicted expression score of *AIM2* was associated with an increased risk of CVD events in patients with *JAK2* and *ASXL1* CH (Figures 3 and 4). While AIM2 inflammasome activation has been linked to *JAK2* CH (15, 39), the AIM2 inflammasome has not previously been associated with *ASXL1*. To understand whether *Asxl1* mutations promote AIM2 inflammasome activation, we introduced truncation mutations into mouse hematopoietic stem and progenitor cells (HSPCs) in exon 12 of *Asxl1* using CRISPR (Figure 5A). Bone marrow–derived macrophages (BMDMs) from mice with CRISPR guides (control) or *Asxl1* mutations (Asxl1-G623*) showed no genotype-dependent alteration in NLRP3 inflammasome activation when challenged with LPS and ATP (Figure 5B). In contrast, *Asxl1*-mutant macrophages demonstrated a selective increase in AIM2 inflammasome activation when treated with the double-stranded DNA fragments (pdAdT) (Figure 5B). Consistent with increased inflammasome activation, *Asxl1*-mutant macrophages had increased LPS-induced *Il1b* production without altered *Casp1* or *Il1rap* expression (Figure 5, C–E). LPS-induced *Nlrp3* expression was reduced in *Asxl1*-mutant macrophages (Figure 5F), which may explain why we did not observe increased NLRP3 activation even in the presence of increased *Il1b*. *Aim2* expression was unchanged in *Asxl1*-mutant macrophages (Figure 5G). Since the AIM2 inflammasome may be activated in response to DNA damage, we measured p-γ-H2AX, a marker of nuclear DNA damage and double-strand break formation (40), and found a significant increase in p-γ-H2AX in *Asxl1*-mutant cells (Figure 5, H and I). These observations suggest that *Asxl1* mutant macrophages have increased *Il1b* expression and increased DNA damage that together lead to increased AIM2 inflammasome activation.

### Asxl1-mutant macrophages have pro- and antiinflammatory characteristics.

**Figure 6:**
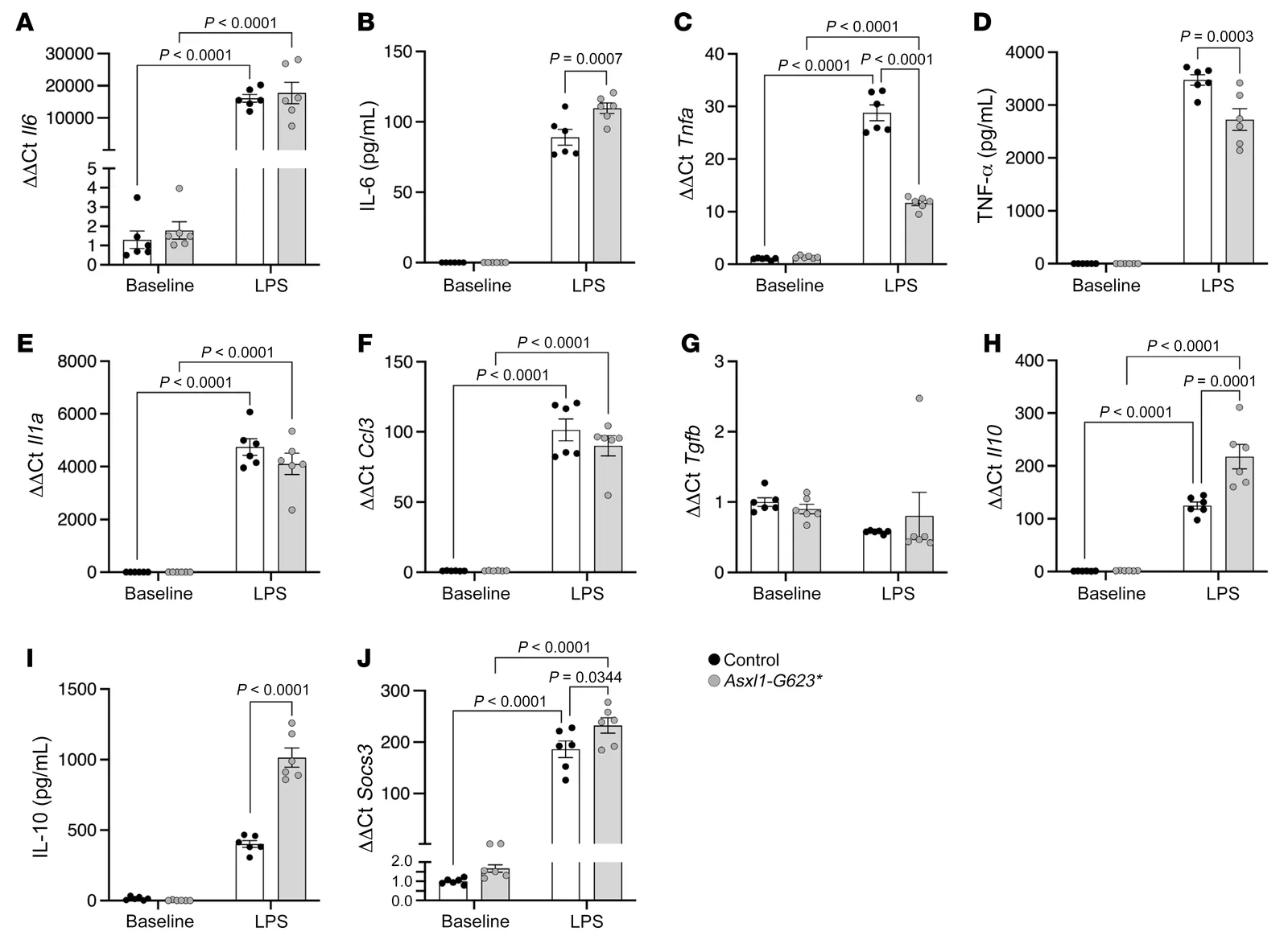
*Asxl1*-mutant macrophages have pro- and antiinflammatory characteristics. BMDMs were untreated (baseline) or treated with 20 ng/mL LPS for 6 hours. (**A**) qPCR analysis. (**B**) ELISA quantification of protein in culture media. (**C**) qPCR analysis. (**D**) ELISA quantification of protein in culture media. (**E**–**H**) qPCR analysis. (**I**) ELISA quantification of protein in culture media. (**J**) qPCR analysis. Data are mean ± SEM. Two-way ANOVA followed by Tukey’s post hoc test, **A**–**J**.

Although AIM2 inflammasome activation has been shown to be sufficient to promote atherosclerosis in *Jak2* CH (15), our findings suggest that other pathways may also contribute to *ASXL1*-mediated CVD risk (Figure 3 and 4). Therefore, we examined inflammatory mediators secreted by BMDMs under baseline and LPS-stimulated conditions. In response to LPS, *Asxl1*-mutant macrophages had no change in *Il6* expression; however, IL-6 secretion was increased (Figure 6, A and B), which is consistent with elevated IL-6 in serum from patients with *ASXL1* CH (16). Interestingly, *Tnfa* expression and secretion were both reduced in *Asxl1*-mutant macrophages (Figure 6, C and D), while we did not see a similar suppression of other LPS-sensitive genes, such as *Il1b*, *Il6*, *Il1a*, *Ccl3*, and *Tgfb* (Figure 5C and Figure 6, A, E, and F). These observations suggest that although LPS-induced inflammatory signaling was largely intact in *Asxl1*-mutant macrophages, some components may be disrupted, potentially due to *Asxl1*-mediated changes in chromatin accessibility. We found that the predicted expression of *IL10* may play a protective role in *ASXL1*-mediated CVD risk in humans (Figures 3 and 4), and IL-10 is also a potent inhibitor of TNF-α. Therefore, we examined whether IL-10 was dysregulated in the presence of *Asxl1* mutations. We observed that stimulation with LPS increased expression of the antiinflammatory mediator *Il10* more than 2-fold in *Asxl1*-mutant BMDMs compared to control and resulted in a similar increase in secreted IL-10 (Figure 6, H and I); this was paralleled by an increase in the *Il10* target gene *Socs3* (Figure 6J). *Socs3* was also found to be increased in *Asxl1*-mutant zebrafish (41). Thus, our population genetic data identified the predicted expression of *IL10* as a potential suppressor of *ASXL1*-mediated CVD, which is supported by functional studies suggesting that IL-10 levels and signaling are increased in *Asxl1*-mutant macrophages and may play an important role in inflammation regulation.

### Asxl1 mutations and atherosclerosis.

**Figure 7:**
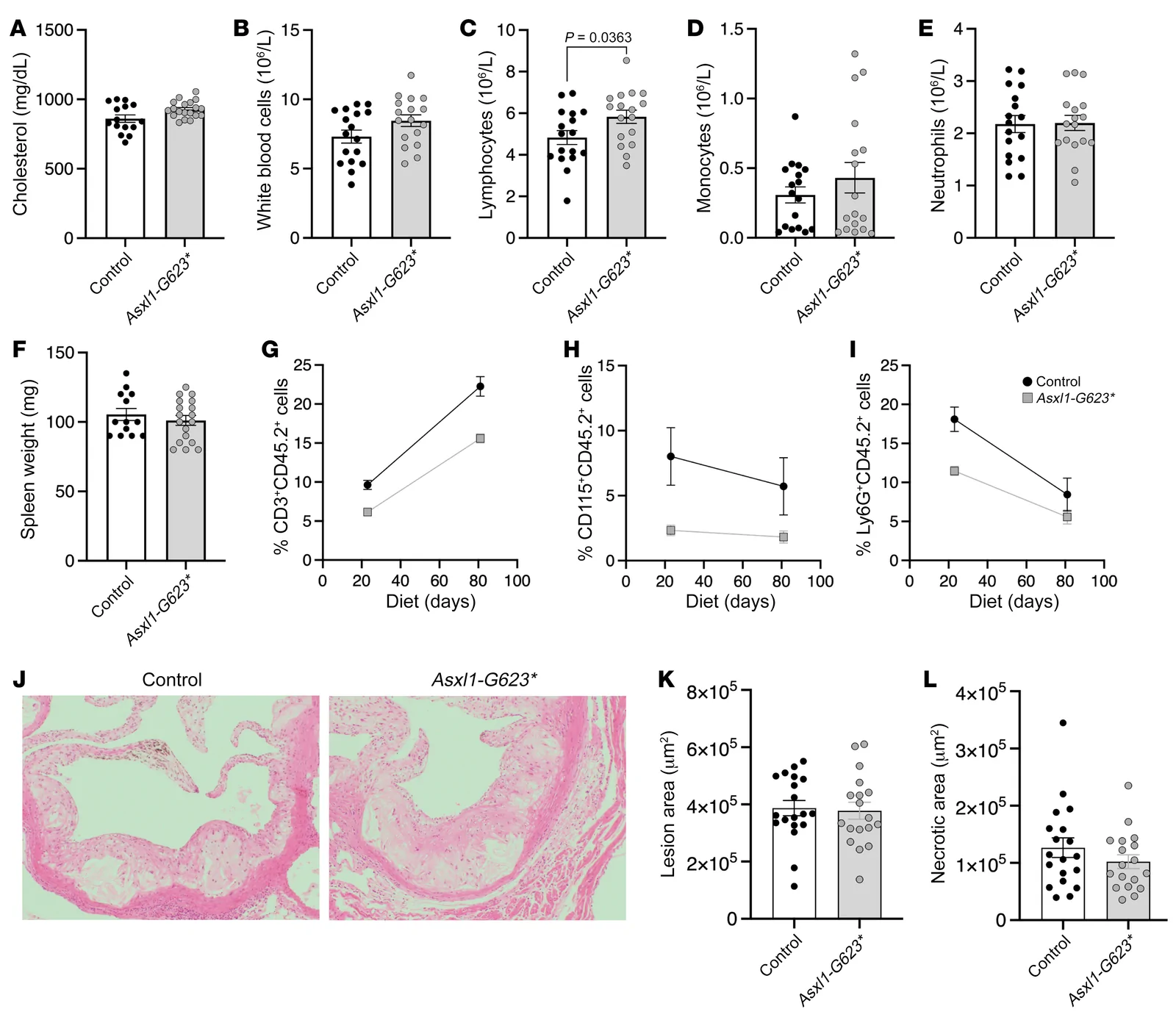
*Asxl1* mutations and atherosclerosis. Mice receiving transplants of chimeric mixtures of bone with nontargeting guide RNAs (control) and Asxl1-G623* guides. (**A**) Terminal serum cholesterol. Complete blood cell counts at the end of WTD feeding for (**B**) white blood cells, (**C**) lymphocytes, (**D**) monocytes, and (**E**) neutrophils. (**F**) Spleen weight. %CD45.2^+^ mutated cells in blood (**G**) lymphocytes, (**H**) monocytes, and (**I**) neutrophils. (**J**) Representative images of H&E-stained aortic root lesions, and (**K**) quantification of lesion area and (**L**) necrotic core area. Data are mean ± SEM. Students *t* test, **A**–**F**, **K**, and **L**. Two-way ANOVA followed by Tukey’s post hoc test, **G**–**I**. Magnification in **J** is ×10.

To determine the impact of *Asxl1* on atherosclerosis, we attempted to model *Asxl1* CH by transplanting CD45.2^+^Cas9^+^ transgenic long-term hematopoietic stem cells (LT-HSCs) infected with control (nontargeting guide RNAs) or Asxl1-G623* guide RNAs mixed with CD45.1^+^ WT cells into lethally irradiated *Ldlr^–/–^* mice. We then placed mice on a Western-type diet (WTD) to induce hypercholesteremia (Figure 7A). *Asxl1* mutations did not alter leukocyte counts in blood or spleen weight (Figure 7, B–F). *Asxl1*-mutant blood cells made up only approximately 15% of lymphocytes, 5% of neutrophils, and 2% of blood monocytes by the end of the study (Figure 7, G–I), indicating a very low mutation burden in these animals. Histological analysis of aortic root lesions indicated no change in the lesion area or necrotic core area (Figure 7, J–L). Our current observations are consistent with previous reports showing impaired initial HSC proliferation and clonal expansion in *Asxl1* CHIP mice and suggest that a much longer follow-up time (>1 year) may be needed to promote atherosclerosis development in the *Asxl1* mouse model (42).

### Associations with hematopoietic traits and cardiometabolic biomarkers.

We examined the associations between the 8 CHIP mutation–predicted gene expression score pairs that had shown significant modification of CVD incidence in our study and 31 hematopoietic traits and 5 common cardiometabolic biomarkers among participants with the corresponding CHIP mutations. After accounting for multiple-hypothesis testing (*n* = 248 [8 × 31] for hematopoietic traits and *n* = 40 [8 × 5] for cardiometabolic biomarkers), we did not observe any significant associations achieving a value below the FDR threshold of 0.05. The suggestive nominal associations were observed between the predicted expression score of *IL18RAP* and reduced eosinophil count and eosinophil percentage among individuals with *ASXL1* mutations (*P* = 0.002 and *P* = 0.003, respectively). This is in line with previous cap analysis of gene expression (CAGE) sequencing data showing that *IL18RAP* is highly expressed in eosinophils, neutrophils, and NK cells (43) (Supplemental Figure 2 and Supplemental Tables 5 and 6).

## Discussion

Leveraging validated human genetic instruments, we showed that specific inflammatory genes may influence incident CVD risk in a manner that is specific to the presence of mutations in key CHIP genes. Our observations are consistent with the notions that reduced *AIM2* expression could specifically mitigate *JAK2* mutation–associated CVD risk and that IL1RAP is a key molecule for CHIP-associated CVD risk across multiple CHIP genes — findings in agreement with prior murine studies. Furthermore, we discovered that modification of *AIM2* expression could affect *ASXL1*-associated CVD risk in humans, and corroborated this finding in CRISPR-induced *Asxl1*-mutated murine BMDMs. Our observations provide human genetic and preclinical support toward precision-medicine paradigms for CVD that we believe merit assessment in prospective studies.

Our study has 3 key implications. First, our findings further show that CVD prognosis and mechanism are distinguished according to the implicated CHIP gene. Prior studies showed that NLRP3 inflammasome inhibition mitigates the heightened atherogenesis observed in *Tet2*-chimeric atherogenic mice compared to atherogenic mice WT for *Tet2* (11). Correspondingly, a common disruptive coding variant in *IL6R* (a downstream mediator of NLRP3) modifies *TET2* or *DNMT3A*-associated CVD risk among humans (7, 45). A post hoc exploratory analysis of a completed clinical trial of a monoclonal antibody targeting IL-1B (also a downstream mediator of NLRP3) supports this finding (45). Recently, it was observed that atherogenic mice expressing *Jak2^VF^* displayed a 2-fold increase in atherosclerotic lesion area with increased features of plaque instability that were reduced in the presence of hematopoietic *Aim2* deficiency. The present study used human genetics as an instrument and observed similar attenuation effects, with genetically predicted lower expression levels of *AIM2* on *JAK2*-associated CVD risk. These data lend support for addressing *JAK2*-associated increased CVD risk through AIM2 inflammasome inhibition.

Furthermore, we discovered AIM2’s potential modulatory role for *ASXL1*-associated CVD risk in humans and validated this by demonstrating increased AIM2 inflammasome activation in BMDMs harboring CRISPR-induced *Asxl1* mutation. In contrast, *Asxl1* mutations did not alter NLRP3 inflammasome activation, which is implicated in *TET2*-associated CAD (11). We further explored the underlying mechanisms. Prior studies showed that *Asxl1*-mutant knockin mice had elevated reactive oxygen species and increased DNA damage (42), and our work further linked the induced DNA damage to AIM2 inflammasome activation. Regarding the proposed mechanism, we noted that mutated *ASXL1* formed a complex with BAP1, leading to enhanced histone deubiquitylation activity. Given the well-documented role of BAP1 in the DNA damage response through posttranslational modifications of histones (46, 47), it is likely that binding of BAP1 to mutated *ASXL1* may suppress the DNA damage response pathway, causing double-strand DNA breaks to accumulate.

Second, our *Asxl1*-mutant macrophage experiments demonstrated both pro- and antiinflammatory properties, a feature of *Asxl1* that has been previously reported in zebrafish by Avagyan et al. (41). Our study revealed a complex expression profile in *Asxl1*-mutant macrophages, potentially linked to alterations in chromatin architecture due to direct histone modifications by *ASXL1* (48). Although we noted an increase in IL-6 secretion, our results also demonstrated a decrease in *Tnfa* expression and secretion. Concurrently, we found an increase in expression and secretion of *Il10*, a *Tnfa* inhibitor, in *Asxl1*-mutant macrophages in murine models. Concordantly, increased predicted *IL10* expression was associated with reduced CVD risk in *ASXL1* CHIP. Together these findings could indicate an important antiinflammatory role for IL-10 expression linked to suppression of CVD in *ASXL1* CHIP.

Third, we observed that increased genetic predisposition to *IL1RAP* expression yielded increased incident CVD risk for participants with *DNMT3A* or *JAK2* CHIP mutations. IL-1RAP is a transmembrane protein that potentiates multiple inflammatory signaling pathways, including IL-1, IL-33, IL-36G, and stem cell factor (27, 28), and it has the unique feature of being expressed at higher levels in stem and progenitor cells from myeloid leukemia patients compared to normal HSPCs (49–52). These properties of IL-1RAP led to several studies investigating the targetability of IL-1RAP as a treatment strategy for myeloid leukemia (25, 51, 53, 54) and may underlie its modification of CHIP-associated CVD and, potentially, other disease risks. These observations agree with the aforementioned human genetic observations using a common missense variant in *IL6R* (7). Furthermore, *Dnmt3a*-inactivated lineage-negative bone marrow cells versus WT cells transplanted into mice had greater IL-6 concentrations (55), and humans with *DNMT3A* mutations had greater expression of NLRP3-related cytokines among PBMCs (18). While the results above and a prior murine study support the role of AIM2 in *JAK2* CHIP, IL-1β inhibition was shown to also influence indexes related to plaque stability in *Jak2*^VF^ transgenic mice (15). Given the significant impact of predicted IL-1RAP expression across all CHIP-associated CAD risks, whether IL-1RAP represents a more effective therapeutic target than individual inflammasomes or their downstream effectors warrants further study.

Finally, our approach of using genetically predicted expression as a therapeutic instrument in humans can potentially advance precision medicine for CVD and beyond. Precision medicine aims to identify and implement therapies that are maximally efficacious based on key features (56). We leveraged prior insights showing the value of human genetics for therapeutic development prioritization (19). Prior studies have similarly used genotype-imputed transcriptomics to nominate therapeutic targets (57–59). Given the overall relatively low heritability of inflammatory gene expression, we used both summary and individual-level training data to impute gene expression perturbations from human genetics. We now compared effects by strata to identify subgroups that may clinically benefit to the greatest extent from inflammation modulation. Our subsequent murine validation lends overall support to this framework.

Our study has important limitations. First, the predicted expression scores for inflammatory genes are genetic proxies for expression levels from birth, which is well before the acquisition of age-related CHIP mutations. Thus, our analyses do not capture the modification effects after CHIP is manifest, which would more closely mimic what was observed in clinical trials. However, our approach was corroborated by modeling in murine macrophages by the introduction of an inflammatory stimulus after a CHIP mutation was introduced. Second, CHIP mutations remain uncommon in the unselected population, so power is limited for interaction analyses. Third, our framework is similarly dependent on suitable heritabilities of the gene expression instruments, and we are thus underpowered to detect associations for instruments with low heritabilities. Since we used individual-level validation data, we were able to exclude instruments with very low heritabilities to optimize multiple-hypothesis testing. Fourth, the majority of participants in our study population — as well as the eQTLGen Consortium, which we used for generating the predicted expression score — were of EA (20, 60); therefore, our findings may not be generalizable to other ancestries. Finally, our computational approaches using human genetics discovered potential modifications of *ASXL1*-associated CVD risk, which was supported by our experiments using *Asxl1*-mutant BMDMs. We set out to model *Asxl1* CH in vivo and monitor atherosclerosis. Yet, in line with other studies (42), we found that introducing *Asxl1* mutations via bone marrow transplantation in mice did not confer a clonal advantage or lead to the development of atherosclerosis within a short time frame. Further research is required to establish a more suitable model before conclusions can be drawn.

In conclusion, in validation of the approach used, our study replicated murine findings in humans indicating that *JAK2* CHIP mutation enhances CVD risk and genetically reduced *Aim2* expression specifically reduced this risk. Examination across other interactions of CHIP variables and predicted expression levels of inflammatory genes on CVD risk yielded additional findings, including modification of *ASXL1*-associated CVD risk by AIM2 expression, which we corroborated in CRISPR-induced *Asxl1*-mutant mouse macrophages. Our results may contribute to developing CHIP type–specific CVD therapies and advance precision medicine goals.

## Methods

### Study population.

In the current analysis, we included the first 424,651 unrelated participants enrolled in the UK Biobank study who underwent exome sequencing of blood DNA and were free of hematologic cancer and CVD at baseline (61, 62). Between 2006 and 2010, approximately 500,000 residents of the United Kingdom (UK) aged 40–69 years were recruited at one of 22 assessment centers across the UK and had samples, including blood-derived DNA, collected at baseline, as well as baseline clinical characteristics, biomarkers, and subsequently incident clinical events through medical history and linkage to data on hospital admissions and mortality. Details regarding this cohort have been described elsewhere in detail (60). Relatedness was defined as one individual in each pair within a third degree of relatedness as determined based on kinship coefficients centrally calculated by UK Biobank (60).

### Whole-exome sequencing and CHIP detection.

Exomes of approximately 450,000 UK Biobank participants were sequenced from blood-derived DNA at the Regeneron Genetics Center, as reported previously (62). Briefly, exomes were captured by Integrated Data Technologies’ (IDT’s) xGen probe library and sequenced on the Illumina NovaSeq platform. Sample-specific FASTQ files were aligned to the GRCh38 reference. The resultant binary alignment file (BAM) containing the genomic information was evaluated for duplicate reads using the Picard3 MarkDuplicates tool and then converted by SAMtools to CRAM files that, after going through quality controls, were submitted to the UK Biobank data repository for distribution. CHIP detection was conducted through using GATK Mutect2 software (https://software.broadinstitute.org/gatk) as previously performed (7, 63, 64). Participants were annotated as having putative CHIP if the output contained at least 1 of a prespecified list of putative CHIP variants in 74 genes anticipated to cause myeloid malignancy at a VAF greater than 2% (Supplemental Table 7) (3, 6, 65). Common sequencing artifacts and germline variants were excluded, as described elsewhere (7).

### RNA-Seq data.

RNA-Seq data were obtained from 2 TransOmics in Precision Medicine (TOPMed) cohorts: MESA and FHS.

MESA is a multiancestry prospective cohort of 6,814 self-identified White, Black, Hispanic, or Asian men and women free of clinical CVD at recruitment in 2000–2002 (66). Included in this study were 889 individuals who had RNA-Seq data in PBMCs measured at baseline. A total of 889 participants were randomly selected from the MESA cohort for RNA-Seq in PBMCs following the standard protocol. For technical details for sample acquisition and RNA-Seq, see Liu et al. (67).

FHS is a multigenerational cohort initiated in 1948 (68). The Framingham Offspring cohort (generation 2 [Gen 2]) was recruited in 1971 (*n* = 5,124), and the Gen 3 cohort was recruited in 2002–2005 (*n* = 4,095) (69, 70). The participants were predominantly self-identified White. Included in this study were 2,622 individuals from the Offspring and Gen 3 cohorts who had peripheral whole-blood samples collected and blood RNA sequenced at exams 9 and 2, respectively. For technical details for the blood draw and RNA-Seq, see Liu et al. (71).

### Gene selection and predicted expression score generation.

We examined pairs of common CHIP mutations that are associated with CVD risk (6), including *DNMT3A*, *TET2*, *ASXL1*, and *JAK2*, and genetically predicted expression levels of inflammatory genes that are biologically closely related to the NLRP3 or AIM2 inflammasomes; these genes were selected based on established biological pathways (72, 73) and protein-protein interactions (74). Specifically, activation of the AIM2 and NLRP3 inflammasomes, both regulated by IFN-γ (72, 75), leads to cleavage of IL-1β and IL-18 to produce their mature forms (76, 77). IL-1β and IL-18 in their active forms then exert diverse biological functions related to inflammation (78), including inducing the production of IL-6, a strong independent predictor of cardiovascular outcomes (79, 80). We therefore included genes encoding these key proteins, namely *IFNG*, *AIM2*, *NLRP3*, *IL1B*, *IL18*, and *IL6R*. Based on the protein-protein interaction networks provided by STRING (https://string-db.org/), we further extended our study to genes that encode proteins with the top 10 highest interaction scores with each of the key proteins (since AIM2 and NLRP3 highly interact, we only kept one of them, NLRP3, as a key protein for selecting genes in the extended list). This resulted in a total of 29 additional genes, namely *CARD8*, *CASP1*, *CASP5*, *DHX33*, *IFNGR1*, *IFNGR2*, *IL10*, *IL18BP*, *IL18R1*, *IL18RAP*, *IL1R1*, *IL1R2*, *IL1RAP*, *IL6*, *IL6ST*, *IRF1*, *JAK1*, *JAK2*, *JAK3*, *NEK7*, *NLRC4*, *SOCS*, *STAT1*, *STAT3*, *STAT4*, *STAT5A*, *STAT6*, *TNF*, and *TYK2*.

For all selected genes, we used genotyping array data from the UK Biobank participants to generate predicted expression scores. The details on quality control and imputation of genotypic data in UK Biobank have been described elsewhere in detail (60). Briefly, genotypic data were obtained using either UK BiLEVE Axiom arrays (Affymetrix Research Service Laboratory) or UK Biobank Axiom and then imputed to either the Haplotype Reference Consortium (HRC) or the merged UK10K+1000 Genomes as reference panel. Principal component analysis (PCA) was performed using fastPCA (81) based on a pruned set of 147,604 single nucleotide variations (SNVs) among unrelated individuals (82).

We calculated the predicted expression score as weighted sums of expression-increase allele counts among selected SNPs, weighted by their raw or posterior effect sizes on the expression levels of the corresponding genes (β coefficient) (22, 83). Raw β coefficient estimates were based on summary statistics of the whole blood (85% of the Consortium) and PBMCs (15% of the Consortium) *cis*-eQTL results from the eQTLGen Consortium (*N* = 31,684; https://www.eqtlgen.org/) (20), with *cis* being defined as within ±500,000 bp around the transcriptional start site (TSS) of the encoding gene of the target protein. The majority of participants included in the eQTLGen Consortium are of European descent, which is similar to our study population (20). We used 2 methods to calculate the scores among EA and non-EA participants separately. (i) One was the pruning + thresholding (P+T) approach, where we used the raw effect size as weights for SNPs and conducted SNPs selection based on the following formula: 


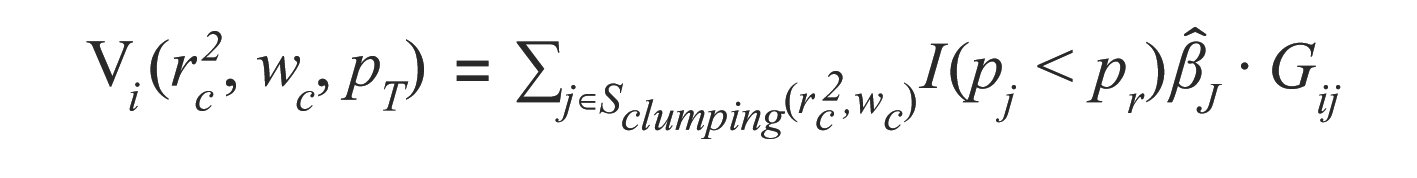


(Equation 1)

where for an individual *i,* 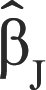 and *p_j_* are the effect size and *P* of variant estimated from the summary statistics, respectively; *G_ij_* is the genotype dosage for that individual *i* and *j* variant; the set of *S_clumping_(r_c_^2^,w_c_)* means restricting to variants remained after clumping at the squared correlation threshold of *r_c_^2^* and clumping window size of *w_c_*; and *I(p_j_ < p_r_)* is a binary indicator function, with 1 indicating *P* of variant *j* less than the specific *P* cutoff *p_r_*, and 0 the other way (21). For each gene, we created 30 candidates’ P+T-based predicted expression scores based on 3 *r*^2^ levels (0.1, 0.01, and 0.001), 5 *P* value thresholds (5 × 10^−8^, 1 × 10^−5^, 0.001, 0.01, and 0.1), and 2 clumping window sizes (within 250 kb and 5 Mb to both ends of the index SNP). (ii) The second method was the PRS-CS approach, which uses a continuous shrinkage Bayesian framework to calculate the posterior mean of effect sizes, used as weights, across all SNPs (22). For each gene, we also created 4 candidate PRS-CS–based predicted expression scores using 4 candidate global shrinkage parameters (1 × 10^−6^, 1 × 10^−4^, 0.01, and 1). For both approaches, we used a set of unrelated individuals from phase 3 of the 1000 Genomes Project as the linkage disequilibrium (LD) reference panel (84). Since eQTLGen summary statistics were from both whole-bold and PBMC samples, we used genotypes and transcriptome concentrations from both FHS (whole blood) and MESA (PBMCs) for score tuning (67). For each gene, we selected the optimal method and parameters for generating the score based on the largest *r^2^* of the measured transcriptome levels in either FHS or MESA, since the eQTL source data were from either whole blood or PBMCs. The best-predicted expression scores were all standardized to zero-mean and unit variance and were approximately normally distributed in the population. In the current study, we continued studying genes whose final-selected best-performed predicted expression scores had *r^2^* > 1% among EA participants, resulting in suitable scores for 26 genes (Figure 2 and Supplemental Table 2).

### Study outcomes.

The primary outcome, CVD event, was a composite of myocardial infarction, coronary artery revascularization, stroke, or death as before (7). We also secondarily used CAD for sensitivity analysis, which was defined as myocardial infarction, percutaneous transluminal coronary angioplasty or coronary artery bypass grafting, chronic ischemic heart disease, and angina. Both disease outcomes were defined by a combination of inpatient hospital billing International Classification of Diseases (ICD) codes and UK death registries, listed in Supplemental Table 8 (7). The exploratory outcomes included 31 hematopoietic cell count indexes and 5 cardiometabolic biomarkers (C-reactive protein [CRP], total cholesterol, HDL cholesterol, LDL cholesterol, and triglycerides). These conventionally measured biomarkers were analyzed as quantitative traits and were log_2_-transformed (with 1 added across all measurements to avoid 0 values for CRP), standardized to zero-mean and unit variance, and normalized in the population. Blood samples of UK Biobank participants were collected into 4 mL EDTA Vacutainers by vacuum draw, stored at 4°C, and then transported to the UK Biocentre in temperature-controlled shipping boxes (85). Full blood counts were measured among all participants using clinical hematology analyzers at the centralized processing laboratory. Serum CRP level was measured by immunoturbidimetric high-sensitivity analysis on a Beckman Coulter AU5800. Lipid measurements were performed on the Beckman Coulter AU5800 platform and run using an immunoturbidimetric approach.

### Asxl1-chimeric mice.

Bone marrow from CD45.2^+^ Cas9 transgenic mice (The Jackson Laboratory, 026179) was harvested and enriched for c-Kit^+^ cells using magnetic beads (Miltenyi Biotec, 130-091-224). LT-HSCs (Lin^–^c-Kit^+^Sca1^+^CD48^–^CD150^+^) (86) were then harvested by flow cytometric sorting. LT-HSCs were then spinfected with 6 μg/mL Polybrene (MilliporeSigma, TR-1003-G) and lentiviruses containing nontargeting guides (*Nmt4*) or guides targeted to *Asxl1* in exon 12 (*Asxl1*-G623*). LT-HSCs were washed and then incubated for 3 days. LT-HSCs were then mixed with 1 × 10^6^ supporting cells from CD45.1^+^ WT mice and transplanted into irradiated *Ldlr^–/–^* recipient mice.

### Asxl1-CRISPR validation.

CRISPR guides targeted to exon 12 of *Asxl1* were designed by CHOPCHOP (87) and screened in skin-derived fibroblasts from Cas9 transgenic mice. Guide sequence AGTGGTAACCTCTCGCCCCTCGG was evaluated by Sanger sequencing of PCR amplification of flanking regions using forward GCAGCATAAAATGGCTCTTGAT and reverse GCTGAGTCTTCTCTTCTGGCTC primers.

### Inflammasome activation studies.

Five weeks after transplantation, bone marrow was harvested and cultured in L cell medium for 5 days to generate BMDMs. 20,000 BMDMs/well were seeded into 96-well plates and allowed to recover overnight. BMDMs were then primed with 20 ng/mL LPS (Cell Signaling Technology, 14011) for 3 hours and stimulated with the indicated concentrations of ATP (MilliporeSigma) for 1 hour. For AIM2 inflammasome activation, BMDMs were primed for 1 hour with 20 ng/mL LPS (Cell Signaling Technology, 14011) then incubated with Lipofectamine 2000 (Thermo Fisher Scientific, 11668019) and poly(deoxyadenylic-deoxythymidylic) acid sodium salt (pdAdT) (Invivogen, tlrl-patn) for 6 hours. Following incubations, supernatants were collected, spun down at 3,000 *g* for 10 minutes, then assessed for IL-1β protein by ELISA (R&D Systems, DY401) and LDH activity (Thermo Fisher Scientific, C20301).

### BMDM cultures.

For protein secretion assays, bone marrow was harvested as indicated above, and after 5 days of differentiation in L cell medium, BMDMs were seeded at 20,000/well in 96 well-plates and allowed to recover overnight. Cells were treated with vehicle (PBS) or LPS at a final concentration of 20 ng/mL for 6 hours. Medium was collected and frozen, and ELISA was conducted to determine concentrations of IL-6 (R&D Systems, DY406), TNF-α (R&D Systems, DY410), and IL-10 (R&D System, DY417).

For mRNA analysis, BMDM were differentiated for 5 days, then seeded into 12-well plates and allowed to recover overnight. Cells were treated with vehicle (PBS) or LPS at a final concentration of 20 ng/mL for 6 hours. BMDMs were then rinsed 3 times with PBS and suspended in TRIzol Reagent (Thermo Fisher Scientific, 15596026), and RNA was isolated using an RNeasy Micro Kit (QIAGEN, 74004) with DNase digestion. cDNA was synthesized (Thermo Fisher Scientific, 4368814), quantitative PCR (qPCR) analysis was conducted, and values were normalized to β-actin expression. Quantification of relative gene expression and percent knockdown were determined using the ΔΔ quantification cycle (*C_q_*) method, derived from *C_q_* values obtained through qPCR analysis. The ΔΔ*C_q_* was computed in a 3-step process. Initially, the *C_q_* values of the gene of interest were normalized to the reference gene, β-actin, using the formula Δ*C_q_* = *C_q_*(gene of interest) – *C_q_*(β-actin). This was followed by an exponential transformation of the expression, denoted as Δ*C_q_* expression = 2^–ΔCq^. Finally, the ΔΔ*C_q_* was calculated by dividing the Δ*C_q_* expression by the average Δ*C_q_* expression of the control group. p–γ-H2AX Western blot analysis was conducted on BMDMs differentiated for 5 days, plated into 6-well dishes, and allowed to recover overnight. BDMDs were treated with the indicated stimulus, including 20 ng/mL LPS, for 6 hours. Cells were then washed 3 times with PBS, and protein was isolated in RIPA buffer (Boston BioProducts, BP-115) with protease and phosphatase inhibitors (Thermo Fisher Scientific, 78439). Protein was quantified with BCA analysis and subjected to Western blotting using antibodies to p–γ-H2AX (Cell Signaling Technology, 9718) and β-actin (Cell Signaling Technology, 12262).

### Atherosclerosis studies.

Bone marrow transplantations were conducted as described above into lethally irradiated *Ldlr^–/–^* mice. After 4 weeks of recovery, mice were subjected to WTD feeding for 12 weeks. Blood cell counts were quantified from cheek bleeding using a VetScan HM5 Hematology system (Abaxis). For *Asxl1* burden analysis, red blood cells were lysed using RBC lysis buffer (BioLegend, 420301), washed in PBS with 1% BSA and 2 mM EDTA, stained with the indicated antibodies (CD3, CD115, Ly6G, CD45.1, and CD45.2), and then analyzed using a LSR-Fortessa. After 12 weeks of WTD feeding, mice were euthanized and perfused with PBS, and aortic roots were fixed in 4% paraformaldehyde for 48 hours. Aortic roots were embedded in paraffin and sectioned 6 μm thick. H&E staining was conducted on 6 slides 60 μm apart and imaged on a Nikon Labophot 2 and Image Pro Plus software (Media Cybernetics, version 7.0.0.591). Researchers blinded to the experimental protocol quantified lesion area and necrotic core area in Fiji software (88), and reported the average for the 6 slides.

### Statistics.

We evaluated the association between CHIP mutations and incident CVD, as well as the modification effects, by predicted expression levels of inflammatory genes measured as predicted expression scores. Using Cox’s proportional-hazard models, we first estimated the HRs and associated 95% CIs of (i) the presence of CHIP mutations and (ii) the presence of large clones, defined as having a VAF > 10%, of CHIP mutations for incident CVD events. Then we conducted stratified analyses evaluating the associations between the predicted expression scores of selected inflammatory genes on the incidence of the primary outcome (i.e., CVD) with or without the presence of CHIP variables. We carried forward predicted expression scores that were associated with incident CVD risk (defined as *P* < 0.05) only in the presence of CHIP variables(s) to evaluate the effect of the interactions between those scores and the corresponding CHIP variables on the primary outcome. We considered time at risk as starting at enrollment in the study and continuing until the event of interest, death, loss to follow-up, or the end of follow-up. Models were adjusted for age at the time of enrollment, sex, self-reported White British ancestry, BMI, diagnoses of type 2 diabetes mellitus at the time of enrollment, ever-smoker status, and the first 10 principal components of genetic ancestry (60). Since only less than 2% of the study population had missingness for any of the adjusted covariates, we removed those individuals from our regression models.

For significant interactions (FDR < 0.05) discovered in the above analysis, we evaluated their associations across 31 hematological and 5 cardiometabolic traits using the same Cox proportional-hazard models with adjustment for the same sets of covariates. All hematological and lipid traits were log_2_-transformed, standardized to zero-mean and unit variance, and were approximately normally distributed in the population. Analyses used R version 4.0.0 software (The R Foundation), 2-tailed *P* values, as well a statistical significance level of 0.05 for other analyses.

### Study approval.

The secondary use of data for the present analysis was approved by the Massachusetts General Hospital Institutional Review Board (protocol 2021P002228) and facilitated through UK Biobank Application 7089. All animal experiments were conducted with approval from the Institutional Animal Care and Use Committee of Columbia University (New York, New York, USA).

### Data availability.

TOPMed individual-level DNA and proteomics data used in this analysis are available with restricted access via the Database of Genotypes and Phenotypes (dbGaP; https://www.ncbi.nlm.nih.gov/gap/). UK Biobank individual-level data are available with request by application (https://www.ukbiobank.ac.uk). Raw data for mouse experiments are reported in the Supporting Data Values file. All code used for the described analyses are available at https://github.com/zhiyu7/chipmodifier (commit ID: 8e634e2).

## Author contributions

ZY, TPF, ART, and PN conceptualized the study. ZY, TPF, YR, ART, and PN developed the methodology. ZY, TPF, and YR performed human statistical analysis and mouse experiments. CV, TN, MMU, TM, AN, JBH, CJG, and GKG conducted CHIP calling. SMZ curated the phenotypes and covariates. YW, GMP, NHC, DL, RSV, FA, KGA, KDT, SSR, and JIR generated and managed the RNA-Seq data used for eQTL score validation. PL, SJ, BLE, AGB, ART, and PN supervised the research. ALT and PN acquired the funding for the research. ZY and TPF wrote the original manuscript draft. YR, CV, TN, MMU, TM, AN, JBH, SMZ, CJG, GKG, YW, GMP, NHC, DL, RSV, FA, KGA, KDT, SSR, JIR, PL, SJ, BLE, AGB, ART, and PN reviewed and edited the manuscript.

## Supplementary Material

Supplemental data

Supporting data values

## Figures and Tables

**Table 2:**
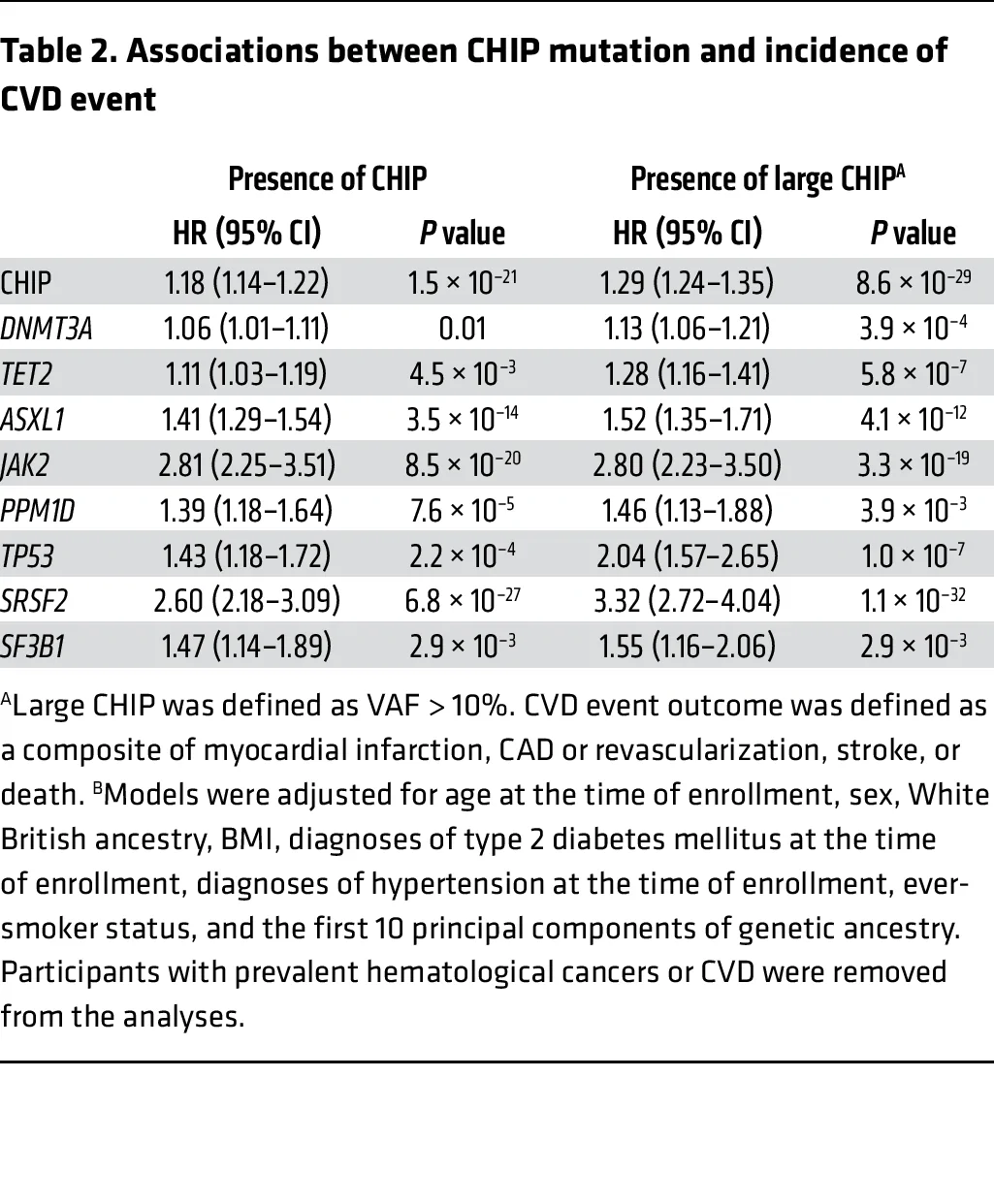
Associations between CHIP mutation and incidence of CVD event

**Table 1:**
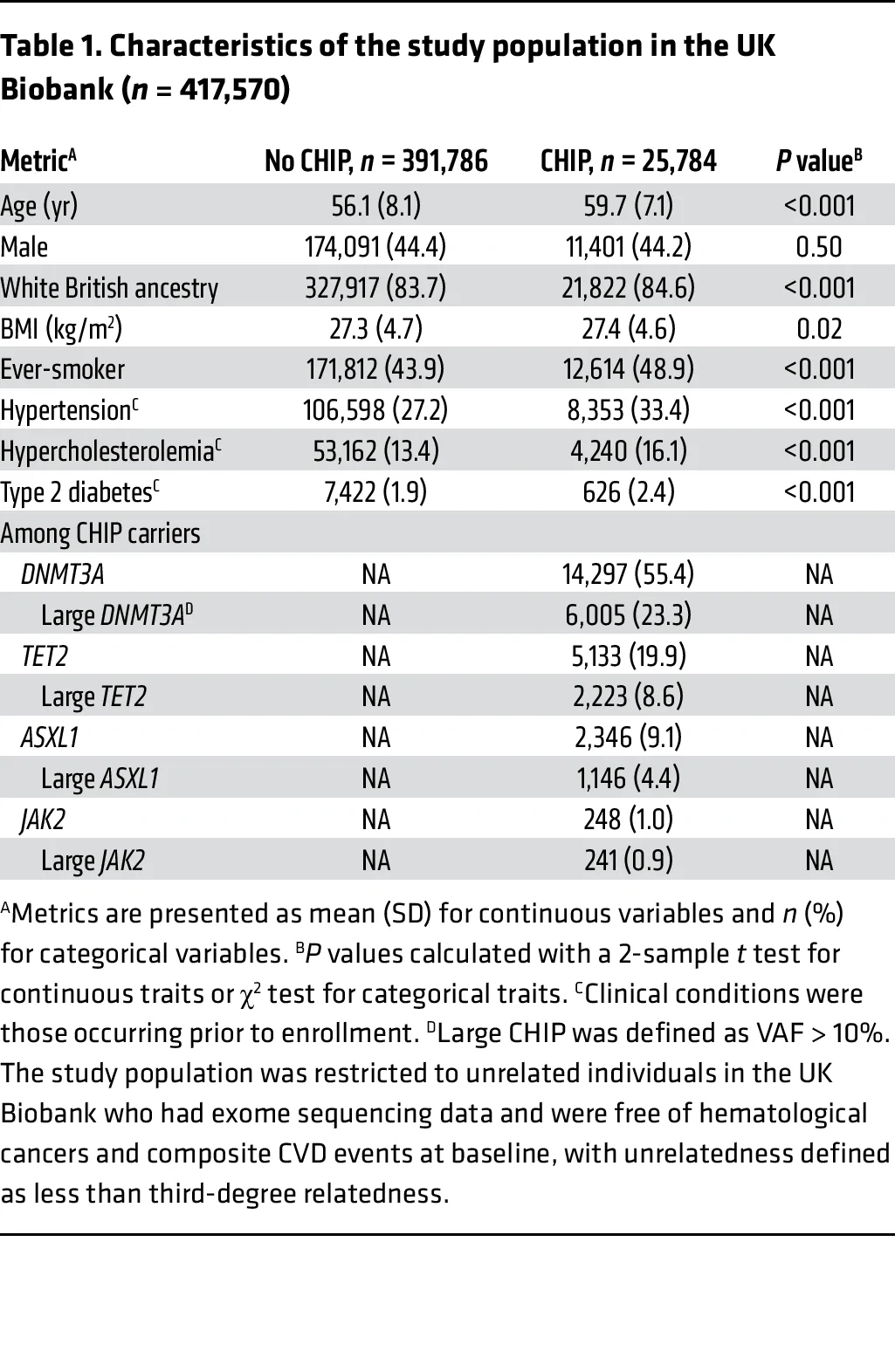
Characteristics of the study population in the UK Biobank (*n* = 417,570)

## Version 1 07/27/2023

In-Press Preview

## Version 2 09/15/2023

Electronic publication

## Version 3 09/29/2026

Corrected supplemental table 3 due to shifted data in ASXL1 (present) column.
