# Corrigendum to Genetic modification of inflammation- and clonal hematopoiesis–associated cardiovascular risk

**DOI:** 10.1172/JCI212049

**Published:** 2026-10-01

**Authors:** Zhi Yu, Trevor P. Fidler, Yunfeng Ruan, Caitlyn Vlasschaert, Tetsushi Nakao, Md Mesbah Uddin, Taralynn Mack, Abhishek Niroula, J. Brett Heimlich, Seyedeh M. Zekavat, Christopher J. Gibson, Gabriel K. Griffin, Yuxuan Wang, Gina M. Peloso, Nancy Heard-Costa, Daniel Levy, Ramachandran S. Vasan, François Aguet, Kristin G. Ardlie, Kent D. Taylor, Stephen S. Rich, Jerome I. Rotter, Peter Libby, Siddhartha Jaiswal, Benjamin L. Ebert, Alexander G. Bick, Alan R. Tall, Pradeep Natarajan

Original citation: *J Clin Invest*. 2023;133(18):e168597. https://doi.org/10.1172/JCI168597

Citation for this corrigendum: *J Clin Invest*. 2026;136(19):e212049. https://doi.org/10.1172/JCI212049

A reader recently identified a discrepancy between Supplemental Table 3 and Figure 3. On review of the original source data, the authors determined that two values in Supplemental Table 3 — the *AIM2* and *CARD8* entries in the *ASXL1* (Present) column — were shifted by one row during table preparation. Figure 3 and the underlying analysis are unaffected. A corrected Supplemental Table 3 is provided in the updated supplemental materials.

The authors regret the errors.

## Version 1 10/01/2026

Electronic publication

