# Supplementary material for: Genetic modification of inflammation- and clonal hematopoiesis–associated cardiovascular risk
Source: J Clin Invest. 2023 Jul 27;133(18):e168597. doi: 10.1172/JCI168597 (PMC10503804; doi:10.1172/JCI168597)
Supplement: Supplementary file 2 [file jci-133-168597-s012.pdf]

Supplemental Table 1. Associations between CHIP mutation and incidence of CAD event. CAD event outcome encompasses individuals with myocardial infarction, percutaneous transluminal coronary angioplasty or coronary artery bypass grafting, chronic ischemic heart disease, and angina. Models were adjusted for age at the time of enrollment, sex, white British ancestry, body mass index, diagnoses of type 2 diabetes mellitus at the time of enrollment, ever-smoker status, and the first ten principal components of genetic ancestry. Participants with prevalent hematological cancers or CAD were removed from the analyses.

	**Presence of CHIP**		**Presence of large CHIP** [a]	
	**HR (95% CI)**	**P-value**	**HR (95% CI)**	**P-value**
**CHIP**	1.05 (1.00, 1.10)	0.05	1.09 (1.02, 1.17)	0.008
DNMT3A	0.97 (0.91, 1.04)	0.37	1.01 (0.91, 1.11)	0.91
TET2	1.02 (0.92, 1.13)	0.67	1.18 (1.03, 1.35)	0.02
ASXL1	1.21 (1.07, 1.38)	0.003	1.16 (0.97, 1.39)	0.10
JAK2	1.52 (1.04, 2.21)	0.03	1.55 (1.06, 2.26)	0.02
PPM1D	1.18 (0.93, 1.49)	0.18	1.14 (0.78, 1.67)	0.49
TP53	1.20 (0.92, 1.56)	0.19	1.51 (1.02, 2.24)	0.04
SRSF2	1.46 (1.09, 1.95)	0.01	1.47 (1.02, 2.10)	0.04
SF3B1	1.21 (0.84, 1.76)	0.30	1.21 (0.79, 1.86)	0.38

[a] Large CHIP is defined as a variant allele fraction >10%.

CHIP, clonal hematopoiesis of indeterminate potential; CAD, coronary artery disease; HR, hazard ratio

Supplemental Table 2. Adjusted proportion of the variance for gene expression level explained by the best-performed predicted expression scores generated using P+T or PRS-CS approaches.

	EUR		Non-EUR	
**Gene**	**Method**	**Variance**	**Method**	**Variance**
IL18RAP	PT	34.70%	PT	3.34%
IL1R1	PT	14.87%	PT	3.78%
TNF	PT	8.97%	PT	1.96%
IL10	PT	8.75%	PT	4.80%
IL1R2	PT	7.48%	PT	3.30%
AIM2	PT	7.33%	PRS-CS	10.96%
IL18R1	PRS-CS	6.36%	PRS-CS	1.29%
CASP5	PT	6.22%	PT	1.63%
TYK2	PT	4.50%	PRS-CS	0.99%
IL18	PRS-CS	4.34%	PRS-CS	3.44%
CASP1	PT	4.27%	PT	2.14%
NLRP3	PT	4.16%	PRS-CS	0.95%
JAK3	PRS-CS	3.57%	PT	2.39%
CARD8	PRS-CS	3.52%	PT	10.82%
NLRC4	PT	3.05%	PT	1.59%
JAK2	PT	3.03%	PRS-CS	2.35%
IFNGR2	PT	2.76%	PRS-CS	5.13%
IFNGR1	PRS-CS	2.04%	PRS-CS	2.23%
IL18BP	PT	1.84%	PRS-CS	0.50%
IL6	PT	1.82%	PT	1.50%
IL1RAP	PRS-CS	1.69%	PT	1.96%
IL6ST	PT	1.53%	PRS-CS	0.55%
STAT4	PRS-CS	1.32%	PT	1.00%
STAT6	PRS-CS	1.32%	PRS-CS	1.55%
NEK7	PT	1.15%	PT	0.58%
IL1B	PT	1.05%	PRS-CS	0.91%

PRS-CS: polygenic risk score-continuous shrinkage; P+T: pruning and thresholding.

Supplemental Table 3. Associations between predicted expression scores of inflammatory genes on incident CVD events stratified by CHIP genes. CVD event outcome is defined as a composite of myocardial infarction, coronary artery disease or revascularization, stroke, or death. Models were adjusted for age at the time of enrollment, sex, white British ancestry, BMI, diagnoses of type 2 diabetes mellitus at the time of enrollment, ever-smoker status, and the first ten principal components of genetic ancestry.

CHIP variables	CHIP		*DNMT3A*		*TET2*		*ASXL1*		*JAK2*	
	Not present (N = 385,678)	Present (N = 25,344)	Not present (N = 396,965)	Present (N = 14,057)	Not present (N = 405,978)	Present (N = 5,044)	Not present (N = 408,719)	Present (N = 2,303)	Not present (N = 410,776)	Present (N = 246)
AIM2	0.99 (0.98, 1.00)[a]	1.00 (0.97, 1.03)	0.99 (0.98, 1.00)	1.00 (0.95, 1.05)	0.99 (0.98, 1.00)	1.00 (0.93, 1.08)	0.99 (0.98, 1.00)	1.14 (1.02, 1.28)	0.99 (0.98, 1.00)	1.85 (1.12, 3.07)
CARD8	1.01 (1.00, 1.02)	0.99 (0.96, 1.02)	1.01 (1.00, 1.02)	0.98 (0.94, 1.02)	1.01 (1.00, 1.02)	1.00 (0.93, 1.07)	1.01 (1.00, 1.02)	0.95 (0.87, 1.03)	1.01 (1.00, 1.02)	1.06 (0.84, 1.34)
CASP1	1.00 (0.99, 1.01)	1.01 (0.98, 1.04)	1.00 (0.99, 1.01)	0.99 (0.95, 1.04)	1.00 (0.99, 1.01)	1.01 (0.94, 1.08)	1.00 (0.99, 1.01)	1.03 (0.94, 1.13)	1.00 (0.99, 1.01)	0.94 (0.72, 1.23)
CASP5	1.00 (0.99, 1.01)	0.99 (0.96, 1.02)	1.00 (0.99, 1.01)	0.98 (0.94, 1.03)	1.00 (0.99, 1.01)	1.00 (0.93, 1.07)	1.00 (0.99, 1.01)	1.03 (0.94, 1.13)	1.00 (0.99, 1.01)	1.08 (0.86, 1.36)
IFNGR1	1.00 (0.99, 1.01)	0.98 (0.95, 1.01)	1.00 (0.99, 1.01)	0.99 (0.95, 1.04)	1.00 (0.99, 1.01)	0.99 (0.92, 1.06)	1.00 (0.99, 1.01)	1.04 (0.95, 1.14)	1.00 (0.99, 1.01)	1.29 (1.01, 1.66)
IFNGR2	1.00 (0.99, 1.01)	1.02 (0.98, 1.05)	1.00 (0.99, 1.01)	1.01 (0.96, 1.06)	1.00 (0.99, 1.01)	1.00 (0.94, 1.08)	1.00 (0.99, 1.01)	1.00 (0.91, 1.10)	1.00 (0.99, 1.01)	0.96 (0.74, 1.25)
IL10	1.00 (0.99, 1.01)	0.99 (0.96, 1.02)	1.00 (0.99, 1.01)	0.99 (0.95, 1.04)	1.00 (0.99, 1.01)	1.03 (0.96, 1.11)	1.00 (0.99, 1.01)	0.91 (0.83, 0.99)	1.00 (0.99, 1.01)	1.01 (0.81, 1.26)
IL18	1.01 (1.00, 1.02)	0.99 (0.96, 1.02)	1.01 (1.00, 1.02)	0.97 (0.93, 1.02)	1.00 (1.00, 1.01)	0.96 (0.90, 1.03)	1.00 (0.99, 1.01)	1.03 (0.95, 1.13)	1.00 (0.99, 1.01)	0.95 (0.75, 1.20)
IL18BP	0.99 (0.98, 1.00)	1.01 (0.98, 1.04)	0.99 (0.99, 1.00)	1.01 (0.97, 1.06)	0.99 (0.99, 1.00)	1.03 (0.96, 1.12)	1.00 (0.99, 1.01)	0.98 (0.89, 1.07)	1.00 (0.99, 1.00)	1.10 (0.80, 1.53)
IL18R1	1.00 (0.99, 1.01)	0.98 (0.95, 1.02)	1.00 (0.99, 1.01)	1.00 (0.95, 1.04)	1.00 (0.99, 1.01)	0.98 (0.91, 1.05)	1.00 (0.99, 1.01)	0.99 (0.91, 1.08)	1.00 (0.99, 1.01)	1.14 (0.90, 1.44)
IL18RAP	1.00 (0.99, 1.01)	0.99 (0.96, 1.02)	1.00 (0.99, 1.01)	1.01 (0.97, 1.06)	1.00 (0.99, 1.01)	1.00 (0.93, 1.07)	1.00 (0.99, 1.01)	0.90 (0.83, 0.98)	1.00 (0.99, 1.01)	1.11 (0.86, 1.42)
IL1B	1.01 (1.00, 1.02)	1.03 (1.00, 1.06)	1.01 (1.00, 1.02)	1.05 (1.01, 1.09)	1.01 (1.00, 1.02)	1.03 (0.97, 1.09)	1.01 (1.00, 1.02)	1.00 (0.91, 1.10)	1.01 (1.00, 1.02)	0.95 (0.69, 1.31)
IL1R1	0.99 (0.98, 1.00)	1.00 (0.97, 1.03)	0.99 (0.98, 1.00)	0.99 (0.95, 1.03)	0.99 (0.98, 1.00)	0.95 (0.88, 1.02)	0.99 (0.98, 1.00)	1.02 (0.93, 1.11)	0.99 (0.98, 1.00)	1.05 (0.82, 1.35)
IL1R2	1.00 (0.99, 1.01)	1.01 (0.98, 1.04)	1.00 (0.99, 1.01)	1.00 (0.95, 1.05)	1.00 (0.99, 1.01)	0.98 (0.91, 1.05)	1.00 (0.99, 1.01)	0.94 (0.86, 1.03)	1.00 (0.99, 1.01)	1.19 (0.88, 1.60)
IL1RAP	1.00 (0.99, 1.01)	1.04 (1.01, 1.07)	1.00 (0.99, 1.01)	1.06 (1.02, 1.11)	1.00 (0.99, 1.01)	1.04 (0.97, 1.11)	1.00 (0.99, 1.01)	0.97 (0.89, 1.07)	1.00 (0.99, 1.01)	1.38 (1.13, 1.69)
IL6	1.00 (0.99, 1.01)	0.99 (0.95, 1.02)	1.00 (0.99, 1.01)	1.02 (0.98, 1.07)	1.00 (0.99, 1.01)	0.93 (0.87, 1.00)	1.00 (0.99, 1.01)	0.99 (0.90, 1.08)	1.00 (0.99, 1.01)	0.92 (0.73, 1.16)
IL6ST	1.00 (0.99, 1.01)	1.02 (0.98, 1.05)	1.00 (0.99, 1.01)	1.01 (0.97, 1.06)	1.00 (0.99, 1.01)	1.07 (1.00, 1.15)	1.00 (0.99, 1.01)	1.00 (0.92, 1.10)	1.00 (0.99, 1.01)	1.10 (0.88, 1.38)
JAK2	1.01 (1.00, 1.02)	1.01 (0.98, 1.04)	1.01 (1.00, 1.02)	1.01 (0.96, 1.05)	1.01 (1.00, 1.02)	1.04 (0.96, 1.11)	1.01 (1.00, 1.02)	0.99 (0.91, 1.08)	1.01 (1.00, 1.02)	1.00 (0.81, 1.23)
JAK3	1.00 (0.99, 1.01)	1.00 (0.97, 1.03)	1.00 (0.99, 1.01)	1.03 (0.98, 1.08)	1.01 (1.00, 1.01)	0.96 (0.89, 1.03)	1.00 (0.99, 1.01)	0.99 (0.91, 1.08)	1.00 (0.99, 1.01)	0.96 (0.77, 1.20)
NEK7	0.99 (0.98, 1.00)	1.00 (0.97, 1.03)	0.99 (0.98, 1.00)	1.01 (0.96, 1.06)	0.99 (0.98, 1.00)	0.98 (0.91, 1.06)	0.99 (0.98, 1.00)	0.96 (0.88, 1.06)	0.99 (0.98, 1.00)	1.02 (0.82, 1.26)
NLRC4	0.99 (0.98, 1.00)	0.97 (0.94, 1.01)	0.99 (0.98, 1.00)	0.96 (0.92, 1.01)	0.99 (0.98, 1.00)	1.01 (0.94, 1.09)	0.99 (0.98, 1.00)	0.97 (0.89, 1.05)	0.99 (0.98, 1.00)	0.94 (0.73, 1.22)
NLRP3	1.00 (0.99, 1.01)	1.01 (0.98, 1.04)	1.00 (0.99, 1.01)	1.00 (0.95, 1.04)	1.00 (0.99, 1.01)	1.01 (0.94, 1.08)	1.00 (0.99, 1.01)	1.00 (0.92, 1.10)	1.00 (0.99, 1.01)	1.20 (0.93, 1.54)
STAT4	1.00 (0.99, 1.01)	0.99 (0.96, 1.03)	1.00 (0.99, 1.01)	1.01 (0.96, 1.05)	1.00 (0.99, 1.01)	0.99 (0.92, 1.06)	1.00 (0.99, 1.01)	0.97 (0.88, 1.06)	1.00 (0.99, 1.01)	0.92 (0.72, 1.19)
STAT6	0.99 (0.98, 1.00)	0.97 (0.94, 1.01)	0.99 (0.98, 1.00)	0.98 (0.94, 1.02)	0.99 (0.98, 1.00)	0.98 (0.92, 1.05)	0.99 (0.98, 1.00)	0.97 (0.89, 1.06)	0.99 (0.98, 1.00)	1.02 (0.81, 1.30)
TNF	0.98 (0.97, 0.99)	0.98 (0.95, 1.02)	0.98 (0.97, 0.99)	0.98 (0.93, 1.02)	0.98 (0.97, 0.99)	0.97 (0.90, 1.04)	0.98 (0.97, 0.99)	0.99 (0.91, 1.08)	0.98 (0.97, 0.99)	1.08 (0.84, 1.37)
TYK2	1.00 (0.99, 1.01)	1.00 (0.96, 1.03)	1.00 (0.99, 1.01)	1.01 (0.96, 1.05)	1.00 (0.99, 1.01)	1.04 (0.97, 1.11)	1.00 (0.99, 1.01)	0.99 (0.90, 1.09)	1.00 (0.99, 1.01)	0.98 (0.75, 1.28)

[a] Effect estimates are represented as hazard ratios (95% confidence interval).

CHIP, clonal hematopoiesis of indeterminate potential; CVD, cardiovascular disease

Supplemental Table 4. Z-scores of interactions between predicted expression scores of inflammatory genes and CHIP mutations on incident CVD events. CVD event outcome is defined as a composite of myocardial infarction, coronary artery disease or revascularization, stroke, or death. Models were adjusted for age at the time of enrollment, sex, white British ancestry, BMI, diagnoses of type 2 diabetes mellitus at the time of enrollment, ever-smoker status, and the first ten principal components of genetic ancestry.

	CHIP	***DNMT3A***	***ASXL1***	***JAK2***
AIM2	-	-	2.38	2.21
IFNGR1	-	-	-	1.39
IL10	-	-	-1.98	-
IL18RAP	-	-	-2.36	-
IL1B	-	1.66	-	-
IL1RAP	2.44	2.72	-	2.31

CHIP, clonal hematopoiesis of indeterminate potential; CVD, cardiovascular disease

Supplemental Table 5. Z-scores of associations between predicted expression scores of inflammatory genes and hematopoietic traits and cardiometabolic biomarkers in the presence of CHIP mutations. Traits were analyzed as quantitative traits and log2-transformed, standardized to zero-mean and unit-variance, and normalized in the population. Models were adjusted for age at the time of enrollment, sex, white British ancestry, BMI, diagnoses of type 2 diabetes mellitus at the time of enrollment, ever-smoker status, and the first ten principal components of genetic ancestry.

	IL1RAP **(*DNMT3A* carriers)**	***IL1RAP*** **(CHIP carriers)**	***AIM2*** **(*ASXL1* carriers)**	***IL18RAP*** **(*ASXL1* carriers)**	***IL1RAP*** **(*JAK2* carriers)**	***AIM2*** **(*JAK2* carriers)**
**White blood cell (leukocyte) count**	0.22	1.14	-0.37	-0.29	0.64	0.31
**Red blood cell (erythrocyte) count**	0.17	-0.22	-0.82	-0.06	-1.29	0.45
**Hemoglobin concentration**	1.07	0.38	-1.03	0.42	-0.84	-1.07
**Hematocrit percentage**	1.18	0.26	-0.74	0.13	-0.62	-0.70
**Mean corpuscular volume**	1.48	0.78	0.27	0.31	1.21	-1.39
**Mean corpuscular hemoglobin**	1.04	0.77	-0.07	0.62	0.81	-1.42
**Mean corpuscular hemoglobin concentration**	-0.44	0.20	-0.62	0.74	-0.56	-0.85
**Red blood cell (erythrocyte) distribution width**	-0.67	-0.40	0.23	-0.10	0.59	-0.12
**Platelet count**	0.15	0.12	0.95	-0.52	0.90	0.40
**Platelet crit**	0.13	-0.28	1.20	-0.74	1.09	0.17
**Mean platelet volume**	0.27	-0.66	0.56	0.13	0.16	-0.23
**Platelet distribution width**	-0.22	-0.52	1.13	0.27	0.23	0.72
**Lymphocyte count**	0.20	0.21	-0.78	-0.95	0.09	-0.61
**Monocyte count**	1.06	1.52	0.98	-1.18	-0.35	1.69
**Neutrophil count**	-0.05	0.94	-0.14	0.82	0.57	-0.16
**Eosinophil count**	0.69	0.95	-0.11	-3.04	0.08	-0.91
**Basophil count**	0.08	0.01	0.36	0.32	-1.08	0.41
**Nucleated red blood cell count**	0.20	1.79	-0.50	-0.51	2.15	0.53

**Lymphocyte percentage**	-0.10	-1.01	-0.41	-1.13	-0.53	-0.76
**Monocyte percentage**	0.95	0.81	1.46	-0.67	-0.35	2.02
**Neutrophil percentage**	-0.38	-0.01	0.31	2.18	0.30	-0.71
**Eosinophil percentage**	-0.15	-0.22	0.07	-2.93	-0.26	-0.90
**Basophil percentage**	-0.04	-0.58	1.14	1.53	-1.74	0.07
**Nucleated red blood cell percentage**	-0.35	0.15	-2.63	-0.16	-0.67	0.63
**Reticulocyte percentage**	-0.38	-0.14	0.20	0.15	0.85	-0.26
**Reticulocyte count**	-0.14	-0.13	-1.31	-0.05	0.22	-0.06
**Mean reticulocyte volume**	0.27	0.02	-0.45	-0.20	0.79	-0.98
**Mean sphered cell volume**	1.37	0.77	-0.49	-0.19	1.25	-0.95
**Immature reticulocyte fraction**	-0.26	0.22	0.81	-0.16	0.62	-0.70
**High light scatter reticulocyte percentage**	-0.16	0.12	0.63	0.14	0.96	-0.38
**High light scatter reticulocyte count**	0.24	0.09	0.35	0.20	0.27	-0.29

CHIP, clonal hematopoiesis of indeterminate potential

Supplemental Table 6. Z-scores of interaction terms formed by pairs of predicted expression scores of inflammatory genes and CHIP showed significant modification effects on CVD risk on five cardiometabolic traits. Traits were analyzed as quantitative traits and log2-transformed, standardized to zero-mean and unit-variance, and normalized in the population. Models were adjusted for age at the time of enrollment, sex, white British ancestry, BMI, diagnoses of type 2 diabetes mellitus at the time of enrollment, ever-smoker status, and the first ten principal components of genetic ancestry.

	IL1RAP **(*DNMT3A* carriers)**	***IL1RAP*** **(CHIP carriers)**	***AIM2*** **(*ASXL1* carriers)**	***IL18RAP*** **(*ASXL1* carriers)**	***IL1RAP*** **(*JAK2* carriers)**	***AIM2*** **(*JAK2* carriers)**
**C-reactive protein**	-1.28	-0.34	0.01	-0.18	1.31	1.63
**Cholesterol**	-0.35	-1.13	-0.28	0.01	-0.57	-0.57
**HDL cholesterol**	-1.62	-2.09	0.10	0.12	-1.46	-0.10
**LDL cholesterol**	0.11	-0.59	-0.78	0.17	-0.53	-0.75
**Triglycerides**	0.56	0.97	2.13	0.33	1.14	1.07

CHIP, clonal hematopoiesis of indeterminate potential; HDL, high-density lipoprotein; LDL, low-density lipoprotein

Supplemental Table 7. List of hematopoietic genes and variants queried.

Gene name	Reported mutations used for variant calling
ASXL1	Frameshift/nonsense/splice-site p.358-1541
ASXL2	Frameshift/nonsense/splice-site in p.380-1435
BCOR	Frameshift/nonsense/splice-site
BCORL1	Frameshift/nonsense/splice-site
BRAF	G464E, G464V, G466E, G466V, G469R, G469E, G469A, G469V, V471F, V472S, L485W, N581S, I582M, I592M, I592V, D594N, D594G, D594V, D594E, F595L, F595S, G596R, L597V, L597S, L597Q, L597R, A598V, T599I, V600M, V600L, V600K, V600R, V600E, V600A, V600G, V600D, K601E, K601N, R603*, W604R, W604G, S605G, S605F, S605N, G606E, G606A, G606V, H608R, H608L, G615R, S616P, S616F, L618S, L618W
BRCC3	Frameshift/nonsense/splice-site
CBL	RING finger missense p.381-421
CBLB	RING finger missense p.372-412
CEBPA	Frameshift/nonsense/splice-site
CREBBP	Frameshift/nonsense/splice-site, D1435E, R1446L, R1446H, R1446C, Y1450C, P1476R, Y1482H, H1487Y, W1502C, Y1503D, Y1503H, Y1503F, S1680del
CSF1R	L301F, L301S, Y969C, Y969N, Y969F, Y969H, Y969D
CSF3R	T615A, T618I, truncating c.741-791
CTCF	Frameshift/nonsense, R377C, R377H, P378A, P378L
CUX1	Frameshift/nonsense
DNMT3A	Frameshift/nonsense/splice-site, F290I, F290C, F290S, G293R, L295P, L295Q, L295V, V296G, V296L, V296M, W297C, W297L, W297R, W297G, G298W, G298R, G298E, W306C, P307S, P307R, P307L, P307T, G308D, I310F, I310L, I310S, I310T, S312F, R326G, R326H, R326L, R326C, R326S, V328A, V328D, V328G, F331V, G332R, G332E, S337A, S337L, S337P, V339A, V339M, V339G, L344Q, L344P, L344R, L347P, L347R, L347Q,S352N,Y365C, R366C, R366P, R366H, R366G, A368D, A368T, A368V, I369N, I369S, V372D, L373Q, A376P, A376T, A376V, R379H, R379L, R379C, R379S, D389N, I407T, I407N, I407S, W409R, A410D, A410T, G413V, F414L, F414I, F414S, F414V, F414C, K468R, E477Q, E477K, V483G, R484Q, R484W, C494Y, C497G, C497R, C497Y, G498E, H506R, G511E, C514Y, Q527H, Q527P, D529N, D529V, D531N, D531Y, Y533C, S535F, S535P, C537G, C537R, C540Y, G543A, G543S, G543C, G543D, G543V, L547H, L547P, L547F, L547R, C537Y, M548I, M548L, M548K,

M548R, M548T, G542V,G550R, C554Y, R556K, R556S, R556G, C559R, C559Y, C562Y, V563M, P580L, W581R, W581G, W581C, W581S, C583S, C583Y, C586G, C586R, C586Y, K589N, L595P, R596W, R598Q, R604Q, R604W, P633H, P633L, I634F, I634T, R635G, R635L, R635P, R635W, R635Q, V636A, V636G, V636M, V636L, L637R, L637P, L637Q, S638F, S638P, S638Y, L639R, L639V, L639F, A644T, T645A, G646V, G646E, L647H, L648P, V649G, V649L, V649M, L650V, L650Q, L653W, L653F, I655N, I655T, Q656K, V657A, V657M, V657G, D658V, D658Y, R659C, R659G, R659H, Y660C, Y660N, Y660F, Y660H, Y660D, A662D, S663L, S663W, E664K, V665G, V665L, S669F, S669P, M674V, V675A, V675M, R676L, R676W, R676Q, I681N, I681S, I681M, M682R, Y683D, V684F, G685R, G685E, G685A, D686Y, D686G, D686H, D686V, V687L, D686A, V687F, R688C, R688G, R688H, V690G, V690F, V690D, T691I, I695N, H694Y, H694P, I695F I695T, Q696P,W698C, W698R, W698S G699R, G699S, G699D, G699V, P700L, P700S, P700R, P700Q, P700T, P700A, F701V, D702A, D702G. D702E, D702V, D702N, D702Y, L703P, L703R, L703V, V704A, V704M, V704G, I705F, I705T, I705S, I705N, G706E, G706W, G706R, G706V, G707C, G707D, G707S, G707R, G707V, C710S, C710Y, D712A, L713F, S714C, V716D, V716F, V716I, N717S, N717I, P718L, R720C, R720H, R720G, R720S, K721R, K721T, K721N, Y724C, E725K, G726V, G728D, R729Q, R729W, R729G, R729L, F731C, F731L, F731Y, F731I, F731V, F732del, F732C, F732I, F732S, F732L, F732V, E733G, E733A, E733V, F734L, F734C, F734V, Y735C, Y735N, Y735S, Y735F, Y735H, R736G, R736H, R736C, R736L, R736P, R736S, L737H, L737P, L737V, L737F, L737R, L737P, L738P, L738Q, H739P, A741G, A741V, R742L, R742G, R742P, P743H, P743R, P743L, P743S, R749C, R749L, R749H, R749G, P750R, F751L, F751C, F751I, F751V, F752del, F752C, F752L, F752I, F752V, W753G, W753C, W753L, W753R, W753S, L754P, L754R, L754H, F755S, F755I, F755L, M761I, M761V, G762C, V763G, V763I, K766E, D768E, D768H, D768V, D768Y, I769N, I769S, I769T, I769V, S770L, S770W, S770P, R771G, R771L, R771P, R771Q, F772C, F772I, F772V, L773H, L773I, L773R, L773V, E774A, E774K, E774D, E774G, E774V, S775F, S775P, P777A, P777H, P777L, P777R, P777T, P777S, V778M, I780N, I780S, I780T, D781G, V785M, A787G, A787S, H789Q, R790W, A791V, R792C, R792H, R792S, F794L, F794V, W795S, W795G, W795C, W795L, W795R, G796A, G796C, G796D, G796V, N797D, N797Y, N797H, L798P, L798H, N797K, N797S, P799L, P799A, P799T, P799S, P799R, P799H, G800S, M801I, M801R, M801T, M801V, R803S, R803G, R803K, R803T, R803W, R803M, P804L, P804S, L815Q, H821D, H821P, H821R, K826N, K826T, K826R, S828N, K829R, T835M, N838D, S839P, K841N, K841T, K841Q, Q842E, Q842R, G843S, P849L, M853R, M852L, M852V, D857N, W860R, E863D, E863G, E863K, E863V, F868S, F868L, G869S, G869V, F870V, H873R, Y874C, R879D, M880I,

	M880L, M880V, S881R, S881I, R882H, R882P, R882L, R882C, R882G, R882S, A884P, A884V, R885K, Q886E, Q886R, L889P, L889R, G890D, G890R, G890S, G890V, W893S, V895M, P896L, P898S, V897G, V897D, I898T, R899P, R899L, R899G, R899H, R899C, R899S, L901P, L901R, L901H, L901V, A903P, A903T, P904L, P904Q, P904A, P904R, P904S, L905R, L905P, L905Q, L905V, K906E, E907G, Y908C, Y908D, Y908N, F909C, A910P, A910V, C911R, C911Y
EED	Frameshift/nonsense, L240Q, I363M
EP300	Frameshift/nonsense, VF1148_1149del, D1399N, D1399Y, P1452L, Y1467N, Y1467H, Y1467C, R1627W, A1629V
ETNK1	N244S, N244T, N244K
ETV6	Frameshift/nonsense
EZH2	Frameshift/nonsense, Q62R, N102S, F145S, F145C, F145Y, F145L, G159R, E164D, R202Q, K238E, E244K, R283Q, H292R, P488S, R497Q, R561H, T568I, K629E, Y641N, Y641H, Y641S, Y641C, Y641F, D659Y, D659G, V674M, A677G, A677V, R679C, R679H, R685C, R685H, A687V, N688I, N688K, H689Y, S690P, I708V, I708T, I708M, E720K, E740K
FLT3	V579A, V592I, F594L, FY590-591GD, D835Y, D835H, D835E, del835
GATA1	Frameshift/nonsense
GATA2	Frameshift/nonsense, R293Q, N317H, A318T, A318V, A318G, G320D, L321P, L321F, L321V, Q328P, R330Q, R361L, L359V, A372T, R384G, R384K
GATA3	Frameshift/nonsense/splice-site ZNF domain, R276W, R276Q, N286T, L348V
GNA13	I34T, G57S, S62F, M68K, Q134R, Y145F, L152F, E167D, Q169H, R264H, E273K, V322G, V362G, L371F
GNAS	R201(844) G, R201(844) S, R201(844) C, R201(844) H, R201(844)L, Q227(870)K, Q227(870)R, Q227(870)L, Q227(870)H, R374(1017)C
GNB1	K57N, K57M, K57E, K57T, I80T, I80N
IDH1	R132C, R132G, R132H, R132L, R132P, R132V
IDH2	R140W, R140Q, R140L, R140G, R172W, R172G, R172K, R172T, R172M, R172N, R172S
IKZF1	Frameshift/nonsense
IKZF2	Frameshift/nonsense
IKZF3	Frameshift/nonsense
JAK1	T478A, T478S, V623A, A634D, L653F, R724H, R724Q, R724P, T782M, L783F
JAK2	N533D, N533Y, N533S, H538R, K539E, K539L, I540T, I540V, V617F, R683S, R683G, del/ins537-539L, del/ins538-539L, del/ins540-543MK, del/ins540-544MK, del/ins541-543K, del542-543, del543-544, ins11546-547

JAK3	M511T, M511I, A572V, A572T, A573V, R657Q, V715I, V715A
KDM6A	Frameshift/nonsense/splice-site, del419
KIT	ins503, V559A, V559D, V559G, V559I, V560D, V560A, V560G, V560E, del560, E561K, del579, P627L, P627T, R634W, K642E, V654A, V654E, H697D, E761D, K807R, D816H, D816Y, D816F, D816I, D816V, D816H, del551-559
KRAS	G12D, G12A, G12E, G12V, G13D, G13C, G13Y, G13F, G13R, G13A, G13V, G13E, V14I, L19F, T58I, G60D, G60A, G60V, Q61K, Q61E, Q61P, Q61R, Q61L, Q61H, K117E, K117N, A146T, A146P, A146V
LUC7L2	Frameshift/nonsense/splice-site
MLL	Frameshift/nonsense
MLL2	Frameshift/nonsense
MPL	S505G, S505N, S505C, L510P, del513, W515A, W515R, W515K, W515S, W515L, A519T, A519V, Y591D, W515-518KT
NF1	Frameshift/nonsense
NPM1	Frameshift p. W288fs (insertion at c.859_860, 860_861, 862_863, 863_864)
NRAS	G12S, G12R, G12C, G12N, G12P, G12Y, G12D, G12A, G12V, G12E, G13S, G13R, G13C, G13N, G13P, G13Y, G13D, G13A, G13V, G13E, G60E, G60R, Q61R, Q61L, Q61K, Q61P, Q61H, Q61Q
PDS5B	Frameshift/nonsense/splice-site, R1292Q
PDSS2	Frameshift/nonsense
PHF6	Frameshift/nonsense/splice-site, A40D, M125I, S246Y, F263L, R274Q, C297Y, H302Y, H329L
PHIP	Frameshift/nonsense/splice-site
PPM1D	Frameshift/nonsense, exon 5 or 6
PRPF40B	Frameshift/nonsense/splice-site, P15H, M58I, P405L, P562S,
PRPF8	M1307I, C1594W, D1598Y, D1598N, D1598V (ADD MORE VARS)
PTEN	Frameshift/nonsense, D24G, R47G, F56V, L57W, H61R, K66N, Y68H, C71Y, F81C, Y88C, D92G, D92V, D92E, H93Y, H93D, H93Q, N94I, P95L, I101T, C105F, C105S, D107Y, L112V, H123Y, C124R, C124S, K125E, A126D, K128N, R130G, R130Q, R130L, G132D, I135V, I135K, C136R, C136F, K144Q, A151T, D153Y, D153N, Y155H, Y155C, R159K, R159S, R161K, R161I, G165R, G165E, S170N, S170I, R173C, Y174D, Y177C, H196Y, R234W, G251C, D252Y, F271S, D326G
PTPN11	G60V, G60R, G60A, D61Y, D61V, D61G, Y63C, E69K, E69G, E69D, E69Q, F71L, F71K, A72T, A72V, A72D, T73I, E76K, E76Q, E76M, E76A, E76G, E139G, E139D, N308D, N308T, N339S, P491L, S502P, S502A, S502L, G503V, G503G, G503A, G503E, Q506P, T507A, T507K

RAD21	Frameshift/nonsense/splice-site, R65Q, H208R, Q474R
RUNX1	Frameshift/nonsense/splice-site, S73F, H78Q, H78L, R80C, R80P, R80H, L85Q, P86L, P86H, S114L, D133Y, L134P, R135G, R135K, R135S, R139Q, R142S, A165V, R174Q, R177L, R177Q, A224T, D171G, D171V, D171N, R205W, R223C
SETBP1	D868N, D868T, S869N, G870S, I871T, D880N, D880Q
SETD2	Frameshift/nonsense, V1190M
SETDB1	Frameshift/nonsense, K715E
SF1	Frameshift/nonsense/splice-site, T454M, Y476C, A508G
SF3A1	Frameshift/nonsense/splice-site, A57S, M117I, K166T, Y271C
SF3B1	G347V, R387W, R387Q, E592K, E622D, Y623C, R625L, R625C, R625G, N626D, R630G, H662Q, H662D, T663I, K666N, K666Q, K666T, K666E, K666R, K700E, V701F, A708T, G740R, G740E, G742D, A744P, A745P, K748E, R775P, D781G, E783K, R831Q, L833F, E862K, R957Q
SFRS2	Y44H, P95H, P95L, P95T, P95R, P95A, P107H, P95fs
SMC1A	K190T, R586W, M689V, R807H, R1090H, R1090C
SMC3	Frameshift/nonsense, R155I, Q367E, D392V, K571R, R661P, G662C
STAG1	Frameshift/nonsense/splice-site, H1085Y
STAG2	Frameshift/nonsense/splice-site
SUZ12	Frameshift/nonsense
TET2	Frameshift/nonsense/splice-site, missense mutations in catalytic domains (p.1104-1481 and 1843-2002), D1121Y, D1129Y, C1133R, C1133W, C1135Y, C1135F, C1135W, G1137D, G1137V, E1137K, E1137D, E1141K, E1144K, Y1148C, L1151R, G1152R, A1153T, A1153V, G1154S, C1156Y, V1157M, I1160F, I1160S, R1161G, R1161S, M1164I, E1165K, E1165D, R1167G, R1167K, R1167S, R1167M, L1172R, A1174T, I1175T, V1180D, M1185I, E1186A, G1187S, K1188R, G1192V, C1193Y, C1193W, P1194L, P1194R, I1195V, K1197E, W1198C, W1198R, V1201I, E1207D, L1209P, L1210P, C1211Y, L1212S, V1213M, V1213E, R1214W, R1214Q, R1216Q, H1219D, H1219R, H1219Y, C1221Y, C1221R, C1221S, C1221W, C1221F, L1229R, G1235E, R1235W, L1238V, A1241S, K1243R, K1243N, L1244P, Y1245C, Y1245N, L1248P, L1248R, L1252P, L1252V, G1256C, R1261C, R1261S, R1261H, R1261P, R1262W, C1263F, C1263Y, N1266D, N1266K, N1266H, N1266Y, N1266S, C1271W, C1271S, C1273S, C1273W, C1273R, Q1274P, G1275R, G1275V, G1282R, G1282D, R1283P, S1284F, F1287V, G1288D, G1288V, C1289F, C1289W, S1290L, W1291C, S1292R, M1293I, Y1294C, G1297E, G1297R, C1298Y, C1298S, K1299M, K1299Q, K1299N, F1300V, F1300L, F1300I, S1303G, S1303R, K1310Q, L1311Q, E1318E, L1322Q, L1322P, L1322R, L1326W, L1329P, L1329Q, L1332P,

M1333K, L1340R, L1340P, Y1345D, Y1345C, Q1348K, Q1348R, I1349N, E1352K, A1355V, C1358S, C1358W, R1359C, R1359L, R1359P, R1359H, R1359G, R1359S, L1360R, G1361C, G1361S, G1361D, R1366H, R1366L, R1366C, P1367L, P1367S, P1367R, F1368L, G1370V, G1370R, V1371D, A1373P, A1376V, D1376G, F1377V, F1377I, C1378R, C1378Y, C1378F, H1380Y, H1380L, H1380R, H1380Q, R1380C, H1380D, H1382R, H1382P, R1383G, H1386D, R1387H, N1387S, T1393A, C1395Y, T1397I, L1398R, F1398C, F1398L, L1398H, L1398P, H1401Y, E1401A, N1403S, Q1414R, Q1414H, Q1414K, V1416I, V1417F, P1419R, D1427V, Q1435K, V1438F, G1861E, G1861R, G1861V, A1863V, H1868Y, H1868P, H1868L, G1869W, S1870L, L1872P, I1873T, I1873S, A1876T, A1876V, R1878P, R1878H, E1879Q, E1879G, E1879D, H1881N, H1881L, T1884A, T1884I, P1889L, N1890S, P1894T, P1894R, P1894L, P1894H, R1896G, I1897N, S1898F, V1900F, E1900K, V1900D, Y1902H, Y1902C, Q1903R, A1903V, H1904R, H1904Q, K1905E, T1905A, H1912R, H1912D, H1912Y, G1913D, A1919D, F1922S, Y1923H, H1925Q, K1934N, R1966C, D1981A, S1982Y

TP53 Frameshift/nonsense/splice-site, S46F, G105C, G105R, G105D, G108S, G108C, R110L, R110C, T118A, T118R, T118I, S127F, S127Y, L130V, L130F, K132Q, K132E, K132W, K132R, K132M, K132N, F134V. F134L, F134S, C135W, C135S, C135F, C135G, C135Y, Q136K, Q136E, Q136P, Q136R, Q136L, Q136H, A138P, A138V, A138A, A138T, T140I, C141R, C141G, C141A, C141Y, C141S, C141F, C141W, V143M, V143A, V143E, L145Q, W146C, W146L, L145R, V147G, P151T, P151A, P151S, P151H, P151R, P152S, P152R, P152L, T155P, T155A, V157F, R158H, R158L, A159V, A159P, A159S, A159D, A161T, A161D, Y163N, Y163H, Y163D, Y163S, Y163C, K164E, K164M, K164N, K164P, H168Y, H168P, H168R, H168L, H168Q, M169I, M169T, M169V, E171K, E171Q, E171G, E171A, E171V, E171D, V172D, V173M, V173L, V173G, R174W, R175G, R175C, R175H, C176R, C176G, C176Y, C176F, C176S, P177R, P177L, H178D, H178P, H178Q, H179Y, H179R, H179D, H179Q, R181C, R181Y, R181H, D186G, G187S, P190L, P190T, H193N, H193P, H193L, H193R, L194F, L194R, I195F, I195N, I195T, R196P, V197L, G199V, Y205D, Y205N, Y205C, V203M, Y205H, D208V, R213Q, R213P, F212I, R213L, R213Q, H214D, H214P, H214R, S215G, S215I, S215R, V216M, V217G, Y220N, Y220H, Y220S, Y220C, E224D, I232F, I232N, I232T, I232S, Y234N, Y234H, Y234S, Y234C, Y236N, Y236H, Y236C, M237V, M237K, M237I, C238R, C238G, C238Y, C238W, N239T, N239S, S241Y, S241C, S241F, C242G, C242Y, C242S, C242F, G244S, G244C, G244D, G245S, G245R, G245C, G245D, G245A, G245V, G245S, M246V, M246K, M246R, M246I, N247I, R248W, R248G, R248Q, R249G, R249W, R249T, R249M, P250L, I251N, L252P, I254S, I255F, I255N, I255S, L257Q, L257P, E258K, E258Q, D259Y, S261T, G262D, G262V, L265P, G266R, G266E, G266V, R267W, R267Q, R267P,

	E271K, V272M, V272L, R273S, R273G, R273C, R273H, R273P, R273L, V274F, V274D, V274A, V274G, V274L, C275Y, C275S, C275F, A276P, C277F, C277Y, P278T, P278A, P278S, P278H, P278R, P278L, G279E, R280G, R280K, R280T, R280I, R280S, D281N, D281H, D281Y, D281G, D281E, D281V, R282G, R282W, R282Q, R282P, E285K, E285V, E286G, E286V, E286K, K320N, L330R, G334V, R337C, R337L, A347T, L348F, T377P
U2AF1	D14G, S34F, S34Y, R35L, R156H, R156Q, Q157R, Q157P
U2AF2	R18W, Q143L, M144I, L187V, Q190L
WT1	Frameshift/nonsense/splice-site
ZRSR2	Frameshift/nonsense, E133G, C181F, D185G, C187Y, H191Y, I202N, F239V, F239Y, N261Y, C280R, C302R, C326R, H330R

Supplemental Table 8. Billing codes used to define outcomes.

Outcome	ICD Codes	UK Biobank data-fields
**Primary outcome (any CVD event)**	Any from "MI", "CAD", "Stroke", or "Death" below	Any from "MI", "CAD", "Stroke", or "Death" below
**MI**		42001 (values: 1,2)
**CAD (Revascularization)**	K40.1, K40.2, K40.3, K40.4, K41.1, K41.2, K41.3, K41.4, K45.1, K45.2, K45.3, K45.4, K45.5, K49.1, K49.2, K49.8, K49.9, K50.2, K75.1, K75.2, K75.3, K75.4, K75.8, K75.9	42001 (values: 1,2)
**Stroke**		42007 (values: 1,2)
**Death**		40020 (any value)
**Type 2 Diabetes**	E11, E11.0, E11.1, E11.2, E11.3, E11.4, E11.5, E11.6, E11.7, E11.8, E11.9	20002 (values: 1223)

CVD: cardiovascular disease, MI: myocardial infarction

IL6R

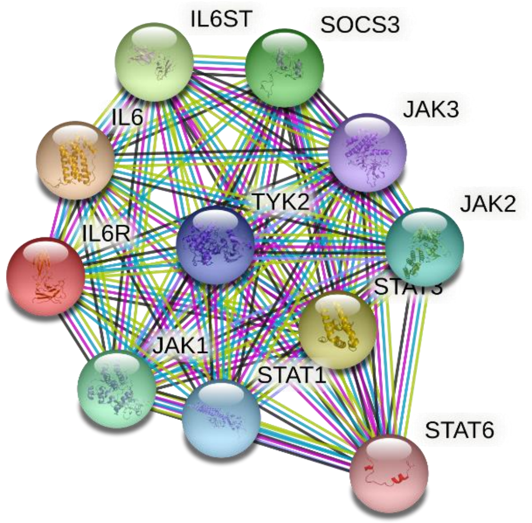

Il1B

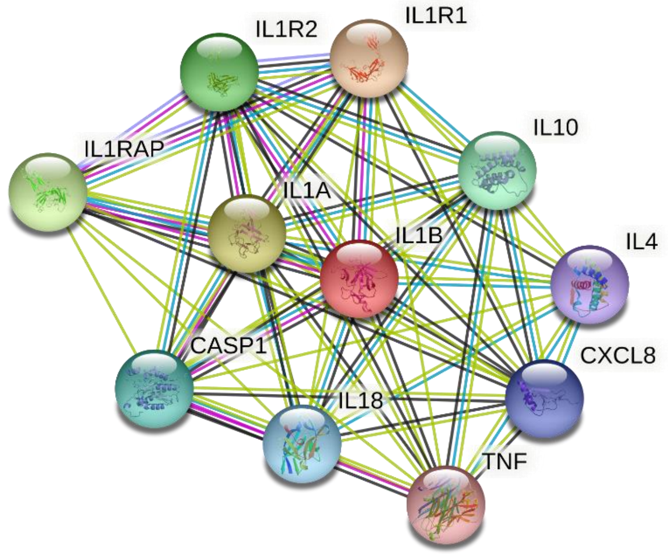

NLRP3

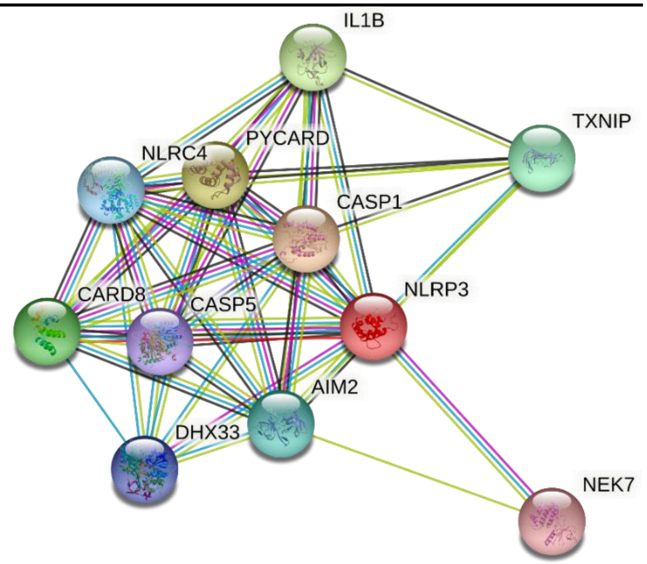

IFNG

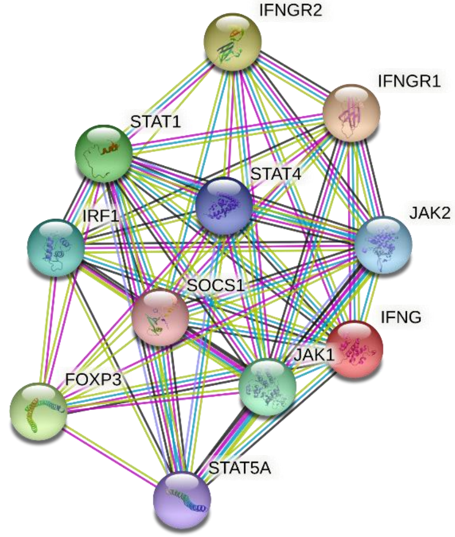

IL18

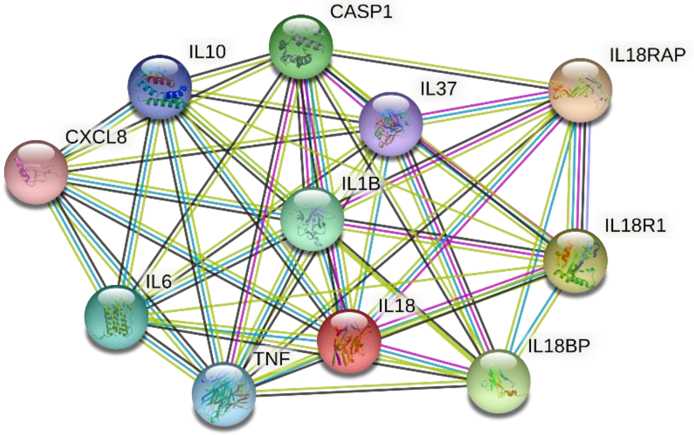

Supplemental Figure 1. Protein-protein interaction network of IL-6R, IL-1B, NLRP3, IFNG, and IL-18. Network structures were obtained from the STRING database (v11; https://string-db.org/). The top ten proteins that interact with IL-6R, IL-1B, NLRP3, IFNG, and IL-18 were presented and selected.

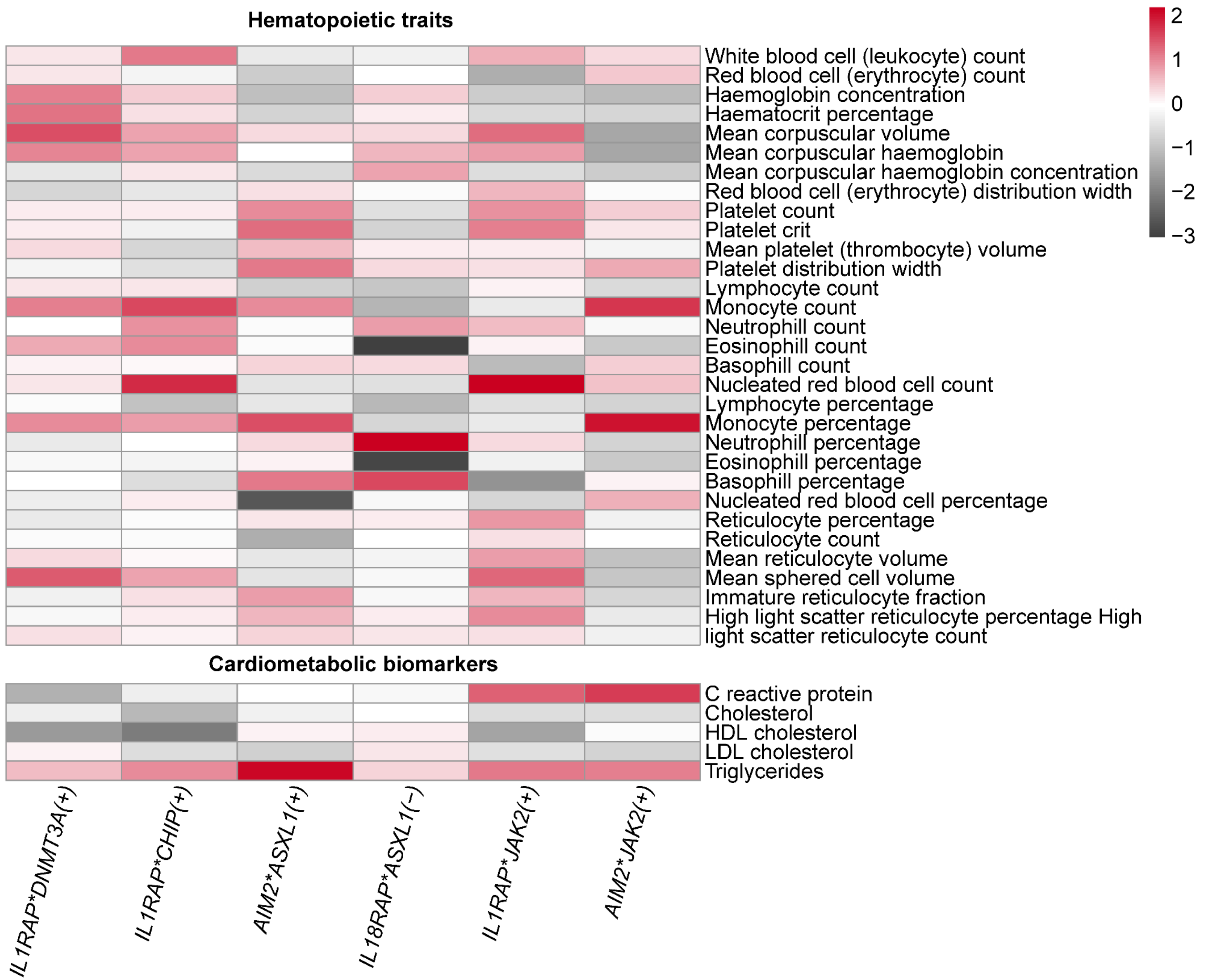
Hematopoietic traits
White blood cell (leukocyte) count
Red blood cell (erythrocyte) count
Haemoglobin concentration
Haematocrit percentage
Mean corpuscular volume
Mean corpuscular haemoglobin
Mean corpuscular haemoglobin concentration
Red blood cell (erythrocyte) distribution width
Platelet count
Platelet crit
Mean platelet (thrombocyte) volume
Platelet distribution width
Lymphocyte count
Monocyte count
Neutrophill count
Eosinophill count
Basophill count
Nucleated red blood cell count
Lymphocyte percentage
Monocyte percentage
Neutrophill percentage
Eosinophill percentage
Basophill percentage
Nucleated red blood cell percentage
Reticulocyte percentage
Reticulocyte count
Mean reticulocyte volume
Mean sphered cell volume
Immature reticulocyte fraction
High light scatter reticulocyte percentage High
light scatter reticulocyte count
Cardiometabolic biomarkers
C reactive protein
Cholesterol
HDL cholesterol
LDL cholesterol
Triglycerides
IL1RAP*DNMT3A(+)
IL1RAP*CHIP(+)
AIM2*ASXL1(+)
IL18RAP*ASXL1(−)
IL1RAP*JAK2(+)
AIM2*JAK2(+)
2
1
0
−1
−2
−3

Supplemental Figure 2. Heatmap for the Z-scores of associations between predicted expression scores of inflammatory genes and hematopoietic traits and cardiometabolic biomarkers in the presence of CHIP mutations. For CHIP mutation-predicted expression score pairs that showed significant interaction for CVD event incidence, we examined the association between the score of inflammatory gene and hematopoietic traits and cardiometabolic biomarkers among participants with the corresponding CHIP mutations. Predicted expression scores of inflammatory genes were calculated by applying either P+T or PRS-CS methods to the summary statistics of the eQTL for those genes from the eQTLGen Consortium (https://www.eqtlgen.org/)**.** Black color indicates a negative Z-score, and red indicates a positive Z-score. No association passed FDR<0.05 or 0.1 level. The darker the color, the stronger the effects. CHIP, clonal hematopoiesis of indeterminate potential. eQTL: expression quantitative trait loci; PRS-CS: polygenic risk score-continuous shrinkage; P+T: pruning and thresholding.

Acknowledgment for individual cohorts in TOPMed

Multi-Ethnic Study of Atherosclerosis:

Whole genome sequencing (WGS) for the Trans-Omics in Precision Medicine (TOPMed) program was supported by the National Heart, Lung and Blood Institute (NHLBI). WGS for "NHLBI TOPMed: Multi-Ethnic Study of Atherosclerosis (MESA)" (phs001416.v1.p1) was performed at the Broad Institute of MIT and Harvard (3U54HG003067-13S1). Centralized read mapping and genotype calling, along with variant quality metrics and filtering were provided by the TOPMed Informatics Research Center (3R01HL-117626-02S1). Phenotype harmonization, data management, sample-identity QC, and general study coordination, were provided by the TOPMed Data Coordinating Center (3R01HL-120393-02S1), and TOPMed MESA Multi-Omics (HHSN2682015000031/HSN26800004). The MESA projects are conducted and supported by the National Heart, Lung, and Blood Institute (NHLBI) in collaboration with MESA investigators. Support for the Multi-Ethnic Study of Atherosclerosis (MESA) projects are conducted and supported by the National Heart, Lung, and Blood Institute (NHLBI) in collaboration with MESA investigators. Support for MESA is provided by contracts 75N92020D00001, HHSN268201500003I, N01-HC-95159, 75N92020D00005, N01-HC-95160, 75N92020D00002, N01-HC-95161, 75N92020D00003, N01-HC-95162, 75N92020D00006, N01-HC-95163, 75N92020D00004, N01-HC-95164, 75N92020D00007, N01-HC-95165, N01-HC-95166, N01-HC-95167, N01-HC-95168, N01-HC-95169, UL1-TR-000040, UL1-TR-001079, UL1-TR-001420, UL1TR001881, DK063491, and R01HL105756. The authors thank the other investigators, the staff, and the participants of the MESA study for their valuable contributions. A full list of participating MESA investigators and institutes can be found at http://www.mesa-nhlbi.org. This study was also supported in part by the National Institutes of

Health, National Heart, Lung, Long and Blood Institute (NHLBI) contract 1R01HL151855, and the National Institute of Diabetes and Digestive and Kidney Diseases contract UM1DK078616.

Framingham Heart Study:

The Framingham Heart Study (FHS) acknowledges the support of contracts NO1-HC-25195, HHSN268201500001I, and 75N92019D00031 from the National Heart, Lung and Blood Institute and grant supplement R01 HL092577-06S1 for this research. We also acknowledge the dedication of the FHS study participants, without whom this research would not be possible. Dr. Vasan is supported in part by the Evans Medical Foundation and the Jay and Louis Coffman Endowment from the Department of Medicine, Boston University School of Medicine.

Acknowledgment for individual cohorts in TOPMed

Multi-Ethnic Study of Atherosclerosis:

Whole genome sequencing (WGS) for the Trans-Omics in Precision Medicine (TOPMed) program was supported by the National Heart, Lung and Blood Institute (NHLBI). WGS for "NHLBI TOPMed: Multi-Ethnic Study of Atherosclerosis (MESA)" (phs001416.v3.p1) was performed at the Broad Institute of MIT and Harvard (3U54HG003067-13S1). Centralized read mapping and genotype calling, along with variant quality metrics and filtering were provided by the TOPMed Informatics Research Center (3R01HL-117626-02S1). Phenotype harmonization, data management, sample-identity QC, and general study coordination, were provided by the TOPMed Data Coordinating Center (3R01HL-120393-02S1), and TOPMed MESA Multi-Omics (HHSN2682015000031/HSN26800004). The MESA projects are conducted and supported by the National Heart, Lung, and Blood Institute (NHLBI) in collaboration with MESA investigators. Support for the Multi-Ethnic Study of Atherosclerosis (MESA) projects are conducted and supported by the National Heart, Lung, and Blood Institute (NHLBI) in collaboration with MESA investigators. Support for MESA is provided by contracts 75N92020D00001, HHSN268201500003I, N01-HC-95159, 75N92020D00005, N01-HC-95160, 75N92020D00002, N01-HC-95161, 75N92020D00003, N01-HC-95162, 75N92020D00006, N01-HC-95163, 75N92020D00004, N01-HC-95164, 75N92020D00007, N01-HC-95165, N01-HC-95166, N01-HC-95167, N01-HC-95168, N01-HC-95169, UL1-TR-000040, UL1-TR-001079, UL1-TR-001420, UL1TR001881, DK063491, and R01HL105756. The authors thank the other investigators, the staff, and the participants of the MESA study for their valuable contributions. A full list of participating MESA investigators and institutes can be found at http://www.mesa-nhlbi.org. This study was also supported in part by the National Institutes of

Health, National Heart, Lung, Long and Blood Institute (NHLBI) contract 1R01HL151855, and the National Institute of Diabetes and Digestive and Kidney Diseases contract UM1DK078616.

Framingham Heart Study:

The Framingham Heart Study (FHS) acknowledges the support of contracts NO1-HC-25195, HHSN268201500001I, and 75N92019D00031 from the National Heart, Lung and Blood Institute and grant supplement R01 HL092577-06S1 for this research. We also acknowledge the dedication of the FHS study participants, without whom this research would not be possible. Dr. Vasan is supported in part by the Evans Medical Foundation and the Jay and Louis Coffman Endowment from the Department of Medicine, Boston University School of Medicine.
